# *Salmonella enterica* serovars in absence of *ttr*A and *pdu*A genes enhance the cell immune response during chick infections

**DOI:** 10.1038/s41598-023-27741-x

**Published:** 2023-01-11

**Authors:** Julia M. Cabrera, Mauro M. S. Saraiva, Lucas B. Rodrigues Alves, Daniel F. M. Monte, Rosemeri O. Vasconcelos, Oliveiro C. Freitas Neto, Angelo Berchieri Junior

**Affiliations:** 1 Sao Paulo State University (Unesp), School of Agricultural and Veterinarian Sciences, Jaboticabal, SP 14884-900 Brazil; 2 Department of Veterinary and Animal Sciences, Faculty of Health and Medical Sciences, University of Copenhagen, 1165 Frederiksberg, Denmark; 3 Federal University of Minas Gerais (UFMG), Veterinary School, Belo Horizonte, MG 31270-901 Brazil

**Keywords:** Microbiology, Infectious diseases, Inflammation

## Abstract

*Salmonella* spp. is one of the major foodborne pathogens responsible for causing economic losses to the poultry industry and bringing consequences for public health as well. Both the pathogen survival ability in the intestinal environment during inflammation as well as their relationship with the host immune system, play a key role during infections in poultry. The objective of this study was to quantify the presence of the macrophages and CD4^+^/CD8^+^ cells populations using the immunohistochemistry technique, in commercial lineages of chickens experimentally infected by wild-type and mutant strains of *Salmonella* Enteritidis and *Salmonella* Typhimurium lacking *ttr*A and *pdu*A genes. *Salmonella* Enteritidis ∆*ttr*A∆*pdu*A triggered a higher percentage of the stained area than the wild-type, with exception of light laying hens. *Salmonella* Typhimurium wild-type strain and *Salmonella* Typhimurium ∆*ttr*A∆*pdu*A infections lead to a similar pattern in which, at 1 and 14 dpi, the caecal tonsils and ileum of birds showed a more expressive stained area compared to 3 and 7 dpi. In all lineages studied, prominent infiltration of macrophages in comparison with CD4^+^ and CD8^+^ cells was observed. Overall, animals infected by the mutant strain displayed a positively stained area higher than the wild-type. Deletions in both *ttr*A and *pdu*A genes resulted in a more intense infiltration of macrophages and CD4^+^ and CD8^+^ cells in the host birds, suggesting no pathogen attenuation, even in different strains of *Salmonella*.

## Introduction

*Salmonella enterica* is a foodborne pathogen that provokes losses to livestock as well as impacts directly on public health. *Salmonella enterica* subsp. *enterica* serovar Enteritidis (*Salmonella* Enteritidis) and Typhimurium (*Salmonella* Typhimurium) have been mainly associated with food infections for decades. Between 1995 and 2010, *Salmonella* Enteritidis was identified in 34,2% of all samples positive for *Salmonella* spp^1^. Besides that, Winter et al.^2^ published a substantial study investigating the tetrathionate-encoding gene role, choosing the serotype *Salmonella* Typhimurium but using mice as an experimental model. Taking this into account, deepening our knowledge of host–pathogen interaction could help to improve control and eradication measurements.

Several factors are involved in salmonellosis pathogenesis, such as the ability of the pathogen to replicate in an inflamed mucous environment, depending on nutrient acquisition and anaerobic respiration^2^. However, nutrient availability does not guarantee bacterial survival in a competitive environment densely populated by other microorganisms. Thus, the ability of *Salmonella enterica* metabolizes tetrathionate employing tetrathionate reductase to produce 1,2-propanediol as an energy source, confers a fitness advantage. This enzyme is constituted by TtrA, TtrB, and TtrC. The first subunit cited belongs to the molybdopterin (MPT) superfamily and has a FeS bounding domain that is involved in the reduction of tetrathionate into thiosulfate (S_2_O_3_^2−^)^2–4^.

The 1,2-propanediol is used by bacterial microcompartments (MCP). This structure is constituted of seven different proteins, among which PduA is the major component of the MCP structure^5^. Firstly, 1,2-propanediol is converted to propionaldehyde, which in turn is reduced to propanol and propionate by propanediol dehydratase activity. This process generates ATP by phosphorylation, an electron (1-propanol) gradient to NAD regeneration, and an intermediary (propionyl-CoA) that can be used as carbon and energy source throughout methyl citrate via, being dependent on B_12_ vitamin synthesized in an endogenous manner^6^.

During chicken infections, the ability of *Salmonella enterica* serovars to invade and survive within the intestinal epithelial cells and macrophages is followed by immune response evasion^7^. In that context, the infection is a critical phase that depends on the interaction between bacterial and host cells and the bacterial ability to overcome the intestinal epithelium barriers to guarantee its colonization and replication. Nevertheless, it activates the inflammatory and immune responses^8^ that lead to endocytosis and phagocytosis by epithelial and antigen-presenting cells (APCs), respectively. The antimicrobial activity of these cells triggers an innate response via macrophages and the adaptive immune response assembly relies on CD4^+^ and CD8^+^ activation^9^.

To shed light on the host–pathogen interactions behind the intestinal infection by *Salmonella* in chickens, we evaluate the population of immune system cells during gut colonization and systemic infection in birds of commercial lineages challenged by wild-type mutant strains of *Salmonella* Enteritidis and *Salmonella* Typhimurium, carrying deletions in genes related to the metabolization of tetrathionate (*ttr*A) and 1,2-propanediol (*pdu*A).

## Results

### Experiment 1—*Salmonella* Enteritidis challenge

The results of quantifying the presence of the immune response cells in caecal tonsils, caecum, ileum, and liver from broiler, light laying hens, and semi-heavy laying hens are shown in Tables 1, 2, and 3. We found more CD4^+^ and macrophages cells infiltration from broilers challenged with *Salmonella* Enteritidis ∆*ttr*A∆*pdu*A (SEΔ*ttr*AΔ*pdu*A) than from broilers challenged with *Salmonella* Enteritidis wild-type strain (wt-SE) or non-infected birds, in all evaluated tissues. An exception was found for CD4^+^ cells infiltration at 3 dpi in the caecal tonsils, ileum, and liver, and for macrophages infiltration at 3 and 14 dpi in the liver from SEΔ*ttr*AΔ*pdu*A challenged broilers. Moreover, the number of CD8^+^ cells infiltrated was observed in a greater quantity from birds challenged with SEΔ*ttr*AΔ*pdu*A at 1 and 7 dpi in the caecum and liver, at 3 dpi in the caecal tonsils, and 14 dpi in the ileum. In contrast, wt-SE-challenged birds had high infiltration of CD8^+^ cells in the ileum and caecal tonsils at 1 and 7 dpi, respectively (Tab. 1; Supplementary Fig. S1).

**Table 1:** Representation of the significant difference related to the quantitative distribution of different immune response cells in organs of broilers infected with *Salmonella* Enteritidis wild and mutant strains at different days post-infection.

Broilers	1 DPI	3 DPI	7 DPI	14 DPI
Caecal tonsils				
CD4^+^	Δ-SE****	wt-SE*	ns	Δ-SE****
CD8^+^	wt-SE*	Δ-SE**	ns	ns
Macrophages	Δ-SE*	Δ-SE****	Δ-SE*	Δ-SE****
Liver				
CD4^+^	Δ-SE**	ns	Δ-SE****	Δ-SE***
CD8^+^	Δ-SE*	ns	Δ-SE****	ns
Macrophages	Δ-SE****	ns	Δ-SE**	ns
Caecum				
CD4^+^	Δ-SE****	Δ-SE****	Δ-SE****	Δ-SE****
CD8^+^	Δ-SE****	Δ-SE****	Δ-SE****	Δ-SE****
Macrophages	Δ-SE****	Δ-SE****	Δ-SE****	Δ-SE****
Ileum				
CD4^+^	Δ-SE****	ns	Δ-SE****	Δ-SE****
CD8^+^	ns	ns	wt-SE***	Δ-SE***
Macrophages	Δ-SE****	Δ-SE****	Δ-SE***	Δ-SE****

DPI, days post-infection; ∆-SE, *Salmonella* Enteritidis ∆*ttr*A∆*pdu*A; wt-SE, *Salmonella* Enteritidis wild-type; ns, no significant difference. Within each organ and DPI, * means difference by two-way ANOVA followed by Bonferroni’s comparison test between wild and mutant strains values (*P ≤ 0.05; **P ≤ 0.01; ***P ≤ 0.001; ****P ≤ 0.0001). The strain presented in the table (∆-SE or wt-SE) as significant within the organ and DPI is that shown the major infiltration area to each cell.

**Table 2:** Representation of the significant difference related to the quantitative distribution of different immune response cells in organs of semi-heavy laying hens infected with *Salmonella* Enteritidis wild and mutant strains at different days post-infection.

Semi-heavy laying hens	1 DPI	3 DPI	7 DPI	14 DPI
Caecal tonsils				
CD4^+^	Δ-SE****	wt-SE***	wt-SE*	wt-SE****
CD8^+^	wt-SE****	ns	ns	Δ-SE*
Macrophages	wt-SE**	ns	Δ-SE*	ns
Liver				
CD4^+^	wt-SE***	ns	Δ-SE*	wt-SE****
CD8^+^	ns	ns	Δ-SE****	Δ-SE**
Macrophages	ns	Δ-SE****	ns	Δ-SE**
Caecum				
CD4^+^	ns	wt-SE**	ns	ns
CD8^+^	ns	Δ-SE*	ns	ns
Macrophages	ns	ns	ns	wt-SE**
Ileum				
CD4^+^	Δ-SE****	ns	ns	Δ-SE****
CD8^+^	wt-SE*	ns	ns	ns
Macrophages	wt-SE****	Δ-SE*	ns	ns

DPI, days post-infection; ∆-SE, *Salmonella* Enteritidis ∆*ttr*A∆*pdu*A; wt-SE, *Salmonella* Enteritidis wild-type; ns, no significant difference. Within each organ and DPI, * means difference by two-way ANOVA followed by Bonferroni’s comparison test between wild and mutant strains values (*P ≤ 0.05; **P ≤ 0.01; ***P ≤ 0.001; ****P ≤ 0.0001). The strain presented in the table (∆-SE or wt-SE) as significant within the organ and DPI is that shown the major infiltration area to each cell.

**Table 3:** Representation of the significant difference related to the quantitative distribution of different immune response cells in organs of light laying hens infected with *Salmonella* Enteritidis wild and mutant strains at different days post-infection.

Light laying hens	1 DPI	3 DPI	7 DPI	14 DPI
Caecal tonsils				
CD4^+^	wt-SE****	wt-SE****	ns	ns
CD8^+^	wt-SE***	ns	ns	ns
Macrophages	ns	wt-SE**	ns	ns
Liver				
CD4^+^	ns	ns	ns	wt-SE*
CD8^+^	ns	ns	ns	ns
Macrophages	ns	ns	ns	ns
Caecum				
CD4^+^	ns	wt-SE***	wt-SE*	ns
CD8^+^	ns	ns	Δ-SE**	ns
Macrophages	ns	wt-SE****	ns	ns
Ileum				
CD4^+^	wt-SE****	ns	wt-SE***	ns
CD8^+^	ns	ns	wt-SE****	wt-SE****
Macrophages	ns	wt-SE****	Δ-SE*	wt-SE****

DPI, days post-infection; ∆-SE, *Salmonella* Enteritidis ∆*ttr*A∆*pdu*A; wt-SE, *Salmonella* Enteritidis wild-type; ns, no significant difference. Within each organ and DPI, * means difference by two-way ANOVA followed by Bonferroni’s comparison test between wild and mutant strains values (*P ≤ 0.05; **P ≤ 0.01; ***P ≤ 0.001; ****P ≤ 0.0001). The strain presented in the table (∆-SE or wt-SE) as significant within the organ and DPI is that shown the major infiltration area to each cell.

Table 2, and Supplementary Fig. S2, show the CD4^+^ and CD8^+^, and macrophage infiltration found in tissues of semi-heavy laying hens. In general, there was great variation between the areas of cell infiltrates concerning both the challenge strain and the tissues studied. Birds challenged with SEΔ*ttr*AΔ*pdu*A showed higher areas covered by CD4^+^ cells (in ileum at 1 and 14 dpi, and liver at 7 dpi), and macrophages (in ileum at 7 dpi, and liver at 3, 7, and 14 dpi), than in the same tissue of wt-SE-challenged birds. On the other hand, CD8^+^ cells were found in larger amounts in caecal tonsils (at 1 and 7 dpi) and caecum (at 3 dpi) of birds challenged with wt-SE strain.

Differently observed in broiler and semi-heavy laying chicks, *Salmonella* Enteritidis ∆*ttr*A∆*pdu*A triggered a less intense immune response cell areas than the wild type, in the challenge from light laying hens. Wt-SE-challenged birds showed larger CD4^+^ infiltration areas in caecal tonsils (at 1 and 3 dpi), caecum (at 3, 7, and 14 dpi), ileum (at 1 and 7 dpi), and liver (at 14 dpi). Similarly, challenges with SEΔ*ttr*AΔ*pdu*A have resulted in reduced infiltration areas of both CD8^+^ in caecal tonsils (at 1 dpi) and ileum (at 7 and 14 dpi), and macrophages in caecal tonsils and caecum (at 3 dpi), in comparison with wt-SE-challenged birds. No significant alterations of immune system cells area of CD8^+^ and macrophage cells were observed in the liver from both SEΔ*ttr*AΔ*pdu*A- and wt-SE-challenged birds. The detailed results of the challenge with light laying hens are shown in Table 3 (see Supplementary Fig. S3).

### Experiment 2—*Salmonella* Typhimurium challenge

The results of quantifying the presence of the immune response cells in cecal tonsils, caecum, ileum, and liver from broilers, semi-heavy laying hens, and light laying hens are shown in Tables 4, 5, and 6, respectively. Overall, broilers infected by the mutant strain displayed a positive marked area higher than those challenged with *Salmonella* Typhimurium wild-type strain (wt-STM) strain, on all four sampling days. Moreover, no alterations of immune response cells were observed in the uninfected control groups.

**Table 4:** Representation of the significant difference related to the quantitative distribution of different immune response cells in organs of broilers infected with *Salmonella* Typhimurium wild and mutant strains at different days post-infection.

Broilers	1 DPI	3 DPI	7 DPI	14 DPI
Caecal tonsils				
CD4^+^	Δ-STM****	ns	Δ-STM*	Δ-STM****
CD8^+^	Δ-STM****	ns	Δ-STM*	Δ-STM***
Macrophages	ns	Δ-STM**	Δ-STM***	ns
Liver				
CD4^+^	Δ-STM****	Δ-STM***	ns	ns
CD8^+^	ns	ns	Δ-STM*	Δ-STM*
Macrophages	Δ-STM***	ns	ns	Δ-STM**
Caecum				
CD4^+^	Δ-STM***	ns	ns	ns
CD8^+^	Δ-STM*	ns	ns	ns
Macrophages	Δ-STM**	ns	ns	Δ-STM*
Ileum				
CD4^+^	Δ-STM****	ns	Δ-STM***	Δ-STM****
CD8^+^	Δ-STM****	ns	Δ-STM***	ns
Macrophages	Δ-STM*	ns	ns	Δ-STM****

DPI, days post-infection; ∆-STM, *Salmonella* Typhimurium ∆*ttr*A∆*pdu*A; wt-STM, *Salmonella* Typhimurium wild-type; ns, no significant difference. Within each organ and DPI, * means difference by two-way ANOVA followed by Bonferroni’s comparison test between wild and mutant strains values (*P ≤ 0.05; **P ≤ 0.01; ***P ≤ 0.001; ****P ≤ 0.0001). The strain presented in the table (∆-STM or wt-STM) as significant within the organ and DPI is that shown the major infiltration area to each cell.

**Table 5:** Representation of the significant difference related to the quantitative distribution of different immune response cells in organs of semi-heavy laying hens infected with *Salmonella* Typhimurium wild and mutant strains at different days post-infection.

Semi-heavy laying hens	1 DPI	3 DPI	7 DPI	14 DPI
Caecal tonsils				
CD4^+^	Δ-STM**	ns	Δ-STM****	Δ-STM**
CD8^+^	ns	ns	ns	ns
Macrophages	Δ-STM**	ns	ns	Δ-STM****
Liver				
CD4^+^	Δ-STM****	ns	Δ-STM*	Δ-STM**
CD8^+^	ns	ns	ns	Δ-STM***
Macrophages	Δ-STM***	ns	ns	Ns
Caecum				
CD4^+^	ns	ns	ns	ns
CD8^+^	Δ-STM****	ns	ns	Δ-STM*
Macrophages	ns	ns	ns	Δ-STM*
Ileum				
CD4^+^	Δ-STM**	ns	ns	ns
CD8^+^	ns	ns	ns	ns
Macrophages	ns	ns	ns	Δ-STM***

DPI, days post-infection; ∆-STM, *Salmonella* Typhimurium ∆*ttr*A∆*pdu*A; wt-STM, *Salmonella* Typhimurium wild-type; ns, no significant difference. Within each organ and DPI, * means difference by two-way ANOVA followed by Bonferroni’s comparison test between wild and mutant strains values (*P ≤ 0.05; **P ≤ 0.01; ***P ≤ 0.001; ****P ≤ 0.0001). The strain presented in the table (∆-STM or wt-STM) as significant within the organ and DPI is that shown the major infiltration area to each cell.

**Table 6:** Representation of the significant difference related to the quantitative distribution of different immune response cells in organs of light laying hens infected with *Salmonella* Typhimurium wild and mutant strains at different days post-infection.

Light laying hens	1 DPI	3 DPI	7 DPI	14 DPI
Caecal tonsils				
CD4^+^	ns	ns	ns	Δ-STM**
CD8^+^	ns	ns	ns	ns
Macrophages	ns	ns	ns	Δ-STM***
Liver				
CD4^+^	Δ-STM****	Δ-STM*	Δ-STM*	Δ-STM**
CD8^+^	Δ-STM****	Δ-STM***	Δ-STM***	Δ-STM***
Macrophages	Δ-STM*	ns	ns	Δ-STM****
Caecum				
CD4^+^	ns	ns	ns	Δ-STM****
CD8^+^	ns	ns	ns	ns
Macrophages	ns	ns	ns	Δ-STM**
Ileum				
CD4^+^	Δ-STM**	ns	ns	Δ-STM*
CD8^+^	ns	ns	ns	ns
Macrophages	Δ-STM***	ns	ns	Δ-STM***

DPI, days post-infection; ∆-STM, *Salmonella* Typhimurium ∆*ttr*A∆*pdu*A; wt-STM, *Salmonella* Typhimurium wild-type; ns, no significant difference. Within each organ and DPI, * means difference by two-way ANOVA followed by Bonferroni’s comparison test between wild and mutant strains values (*P ≤ 0.05; **P ≤ 0.01; ***P ≤ 0.001; ****P ≤ 0.0001). The strain presented in the table (∆-STM or wt-STM) as significant within the organ and DPI is that shown the major infiltration area to each cell.

The CD4^+^ cell areas in all tissues of broilers did reach a statistical difference at 1 dpi, with larger infiltrations for the challenge with *Salmonella* Typhimurium ∆*ttr*A∆*pdu*A (STM∆*ttr*A∆*pdu*A). A significant difference between the area of the quantified immune response from STM∆*ttr*A∆*pdu*A- and wt-STM-challenged birds had been, with larger infiltrations when the challenge was with the mutant strain in the caecum and ileum at 1 dpi, and liver at 14 dpi (CD8^+^ cells); in the caecum, ileum, and liver at 1 and 14 dpi (macrophages) (Table 4; Supplementary Fig. S4).

The results from semi-heavy laying birds showed no statistical difference between mutant and wild-type strain challenges for CD4^+^ cells in the caecum, and CD8^+^ cells in the caecal tonsils and ileum in all 4 days post infections evaluated (Table 5; Supplementary Fig. S5). However, when a significative concentration area of immune system cells was observed the semi-heavy laying hens were challenged by STM∆*ttr*A∆*pdu*A: major macrophages infiltration area in all tissues studied (at 1 and 14 dpi); a major infiltration area of CD4^+^ cells in the caecal tonsils and liver (at 1, 7, and 14 dpi) (Table 5; Supplementary Fig. S5).

Table 6, and Supplementary Fig. S6 show the CD4^+^, CD8^+^, and macrophage infiltration found in the tissues of light laying hens. Birds challenged with STM∆*ttr*A∆*pdu*A showed major immune system cells area of CD4^+^ and macrophage cells in caecal tonsils and caecum (at 14 dpi), and ileum (at 1 and 14 dpi) in comparison to immune response cells area of the same tissues from wt-STM-challenged birds. No statistical difference for the CD8^+^ infiltration area had been found in the caecal tonsils, caecum, and ileum from challenged birds. In contrast to results obtained in other tissues, the liver from STM∆*ttr*A∆*pdu*A-challenged birds had a more expressive stained area of immune response cells for CD4^+^ and CD8^+^ cells at all four dpi, and macrophages at 1 and 14 dpi.

## Discussion

Bacteria, when exposed to anaerobic conditions, may use tetrathionate and 1,2-propanediol metabolic substrates for energy and respiration sources^10^. Thus, *Salmonella* spp. has long been subject to investigations into how the deletion of genes known to be responsible for these pathways would affect their survival in the host. To the best of our knowledge, only one study investigating simultaneously tetrathionate- and propanediol-encodings genes roles was published. Our research group reported the effects of these deletions by evaluating systemic infection and faecal excretion of *Salmonella* Enteritidis and *Salmonella* Typhimurium in commercial lineages of chicks^11^. To increase the discussion on this subject, the present results highlighted the immune cell infiltrated in different tissues of chick lineages challenged with both wild-type and mutant strains carrying deletions in *ttr*A and *pdu*A genes.

Over the 2-week experiment the positively stained areas of CD4^+^ and CD8^+^ cells, and macrophages follow mostly a similar pattern, wherein at 1 and 14 dpi present a higher number of immune response cells. This can be explained by the primary contact of the host defense system when the pathogen invades. The previous report has shown that in chickens infected, even when *Salmonella* is not excreted at 12 dpi, the infection can become positive by cloacal swab from 13 dpi^12^, explaining why immune system cells areas at 3 and 7 dpi were lower but back to an increase.

At first glance, it would be expected that the elicited response of the host would be reduced when both *pdu*A and *ttr*A were deleted since these genes play an important role in the survival during infection by *Salmonella*^2,4,13–15^. However, our results comparing a double mutant lacking both genes showed the opposite, the mutant strains of *Salmonella* Enteritidis and *Salmonella* Typhimurium triggered higher immune response cells than the wild types of strains. A shortest stained area could lead to a high number of colonies on the intestinal tract, corroborating a previous study, wherein *Salmonella* Enteritidis ∆*ttr*A∆*pdu*A and *Salmonella* Typhimurium ∆*ttr*A∆*pdu*A strains were recovered in higher numbers from cloacal swabs than their wild type correlated^11^.

*Salmonella* can behave as an extracellular or an intracellular bacterium, depending on the nutrient repertoire available, and occurs as a switch between intestinal colonization and internalization into host cells^16^. When the bacteria are ingested and killed by macrophages, some peptide fragments are transferred to the surface of the antigen-presenting cell, being encoded by the major histocompatibility complex (MHC), class II. This peptide–MHC II binding stimulates the T CD4^+^ lymphocytes. However, if the bacteria decide to invade the host cell, entering the cytoplasm of the macrophage, the peptide connection with another type of MHC, class I, stimulates T CD8^+^ lymphocyte production^17^.

Interestingly, CD4^+^ and CD8^+^ cells present the same pattern of the macrophages throughout the experiment, even representing different immune responses. Since CD4^+^ and CD8^+^ cells are mainly representing T lymphocytes which are part of the adaptive immune response, the macrophages, are part of the innate immune response^9^. In addition to this, we observed that broilers present more expressive positive marked area than laying hens, confirmed by a previous study where broilers challenged with mutant strains showed, for example, a more invasive intestinal colonization and systemic infection^11^.

Our findings suggest that the immunohistochemistry approach provides interesting information about the behavior of immune response cells on multiple organs of different commercial lineages during infection by *Salmonella enterica* serovars. Moreover, the present study evidence that deleting both genes, even in different strains of *Salmonella*, resulted in bacteria that elicited a higher immune response cell in the host, showing that the pathogen has not been attenuated. We can consider that perhaps *Salmonella* was able to find another survival mechanism becoming even more pathogenic. The utilization of *ttr* and *pdu* operons in consonance with *cob* and *prp* operons has been shown, in the previous study, necessary for anaerobic respiration^16^, leading us to believe that is not only required to delete more genes from each operon^18^, but we also have to ponder deleting this whole set, to reach less pathogenic strains of *Salmonella enterica*.

## Materials and methods

The experiments, performed following relevant guidelines and regulations, were approved by the Ethical Committee on the Use of Animals of Sao Paulo State University (CEUA/Unesp Process—006621/18; on May 10th, 2018), were carried out in the Avian Pathology Laboratory of the Department of Pathology, Theriogenology, and One Health from the School of Agricultural and Veterinary Sciences, Sao Paulo State University (FCAV/Unesp), Jaboticabal, Brazil.

### Bacterial strains and mutant construction

The bacterial strains used here were stored within a cryoprotectant medium compounded by Lysogeny broth (LB; BD DifcoTM, USA) supplemented with 30% of glycerol (Merck, BR—H30402394 228) and storage in an ultra-freezer (− 80 °C) at the Avian Pathology Laboratory from FCAV/UNESP. *Salmonella* Enteritidis P125109 (accession number: AM933172) and *Salmonella* Typhimurium str. 98^19^ were induced to nalidixic acid- and spectinomycin-resistance (Nal^r^Spc^r^) and they provided the genetic background for constructing mutant strains by Lambda-red technique^20^ with minor modifications, described in Saraiva et al.^11^. Mutant bacterias constructed here are identified on the text as SEΔ*ttr*AΔ*pdu*A (*Salmonella* Enteritidis ∆*ttr*A∆*pdu*A) and STMΔ*ttr*AΔ*pdu*A (*Salmonella* Typhimurium ∆*ttr*A∆*pdu*A).

### In vivo experiment

#### Experiment 1—*Salmonella* Enteritidis

Thirty-six 1-day-old chicks from each of three different lineages (broiler, semi-heavy laying hens, and light laying hens), totaling one hundred and eight animals, were obtained from commercial hatcheries. At arrival, the bottom of transport card boxes was examined to confirm the *Salmonella*-free status of the birds^21^, and the animals were housed within metallic cages inside the acclimatized room and received antibiotic-free feed and water ad libitum. A 24-h light program was chosen on the first day to ensure optimal water and food ingestion, then a 12-h light program was adopted in the first week, decreasing to 8 h on the remaining days.

The inoculum was prepared according to Berchieri Junior et al.^22^. For this, the frozen cultures were inoculated in LB and incubated overnight at 37 °C under 150 rpm. On the following day, the bacterial cultures were transferred into fresh media and incubated for 18 h under the same conditions as previously. Then, 0.2 mL from the cultures containing 10^8^ colony-forming units per mL (CFU/mL) were orally inoculated by metallic gavage directly into the birds’ crop.

Nine groups were formed (A to I) and randomly divided according to the different lineages and strains (Table 7). At one-, three-, seven-, and 14-days post-infection (dpi), three birds per group each day, by morning, were euthanized by cervical dislocation to harvest the medial section of caecal tonsils, caecum, and ileum, and the distal section of the liver left lobe for further immunohistochemistry (IHC) analysis. For this, samples were submerged within n-Hexane p.a. (n-Hexano p.a., Synth, Brazil) previously refrigerated in liquid nitrogen. Immediately after the tissue freezing, it was transferred into a 2 mL cryotube (Corning, USA) and conditioned in liquid nitrogen. After sampling, the tissues were stored at − 80 °C until the process for IHC.

**Table 7:** Established groups according to the different lineages and strains.

Group	Lineage	Strain
A	Broiler	SE∆*ttr*A∆*pdu*A or STM∆*ttr*A∆*pdu*A
B	Broiler	wt-SE or wt-STM
C	Semi-heavy laying hens	SE∆*ttr*A∆*pdu*A or STM∆*ttr*A∆*pdu*A
D	Semi-heavy laying hens	wt-SE or wt-STM
E	Light laying hens	SE∆*ttr*A∆*pdu*A or STM∆*ttr*A∆*pdu*A
F	Light laying hens	wt-SE or wt-STM
G	Broiler	NC
H	Semi-heavy laying hens	NC
I	Light laying hens	NC

SE∆*ttr*A∆*pdu*A, *Salmonella* Enteritidis ∆*ttr*A∆*pdu*A; STM∆*ttr*A∆*pdu*A, *Salmonella* Typhimurium ∆*ttr*A∆*pdu*A; wt-SE, *Salmonella* Enteritidis wild-type; wt-STM, *Salmonella* Typhimurium wild-type; NC, negative control.

#### Experiment 2—*Salmonella* Typhimurium

This experiment was carried out following the same characteristics mentioned above in experiment 1. Thirty-six chicks (1 day old) were randomly divided into nine groups (A to I) based on their lineages and strains (Table 7).

### Immunohistochemistry

#### Tissue section

The collected samples were transferred from − 80 °C to cryostat (Leica CM1860, Leica Biosystems Nussloch GmbH, Germany) at − 22 °C where were individually blocked in O.C.T. compound (Tissue-Tek^®^, Sakura Finetek Europe B.V., Netherlands) per 30 min prior to 6 µm section using low profile disposable blades (Leica 819, Leica Biosystems Nussloch GmbH, Germany). It is noteworthy that sections were done at − 22 °C, except liver sections, which were done at − 15 °C. Slides containing three repetitions of the sectioning of each organ per immune cell response marked were prepared, with thinning between each repetition. Using a paintbrush, the tissue section cuts were placed on histological slides pre-treated with poly-l-lysine (Sigma-Aldrich, United Kingdom, Cat no. P4832) e silane (Sigma-Aldrich, USA. Cat no. 440574). The slides were stored at − 20 °C thereafter until IHC staining.

#### Immune cells staining

Firstly, the slides were submerged in 200 mL refrigerated acetone (Acetone P.A.—A.C.S., Synth, Brazil) and incubated at − 20 °C for 10 min. After that, the slides were transferred into a humidity chamber (EasyPath^®^, Brazil) at room temperature for 5 min to dry the samples. The slides were washed thereafter with PBS and a puddle was left for 5 min to avoid tissue dehydration. Then, tissues were submerged in 200 mL of 4% H_2_O_2_ per 10 min in a dark place and washed again with PBS. The area around the tissue sections was dried with absorbent paper and the sample was bypassed by a hydrophobic pen (Dako Pen, Dako Denmark A/S, Denmark). The washing step leaving a puddle was repeated as previously.

The biotin-free kit Mouse and Rabbit Specific HRP/DAB IHC Detection—Micropolymer (Abcam©, USA) was used to stain the immune cells, choosing the Avidin–Biotin Streptavidin Peroxidase Complex (ABC) method. For this, the puddle was removed, and non-specific background color blocker reagent droplets were added to the tissues. The slides were maintained inside the humidity chamber in a dark place for 30 min, the washing step was repeated, and 200 µL of primary antibody (Mouse Anti-Chicken CD4-UNLB; Mouse Anti-Chicken CD8α-UNLB; Mouse Anti-Chicken Monocyte/Macrophage-UNLB, Southern Biotech, USA) diluted in a proportion of 1:200 (v/v) in the antibody diluent reagent (Antibody diluent, Abcam©, USA) was added thereafter. The slides were incubated at 4 °C for 18 h.

**Figure 1:**
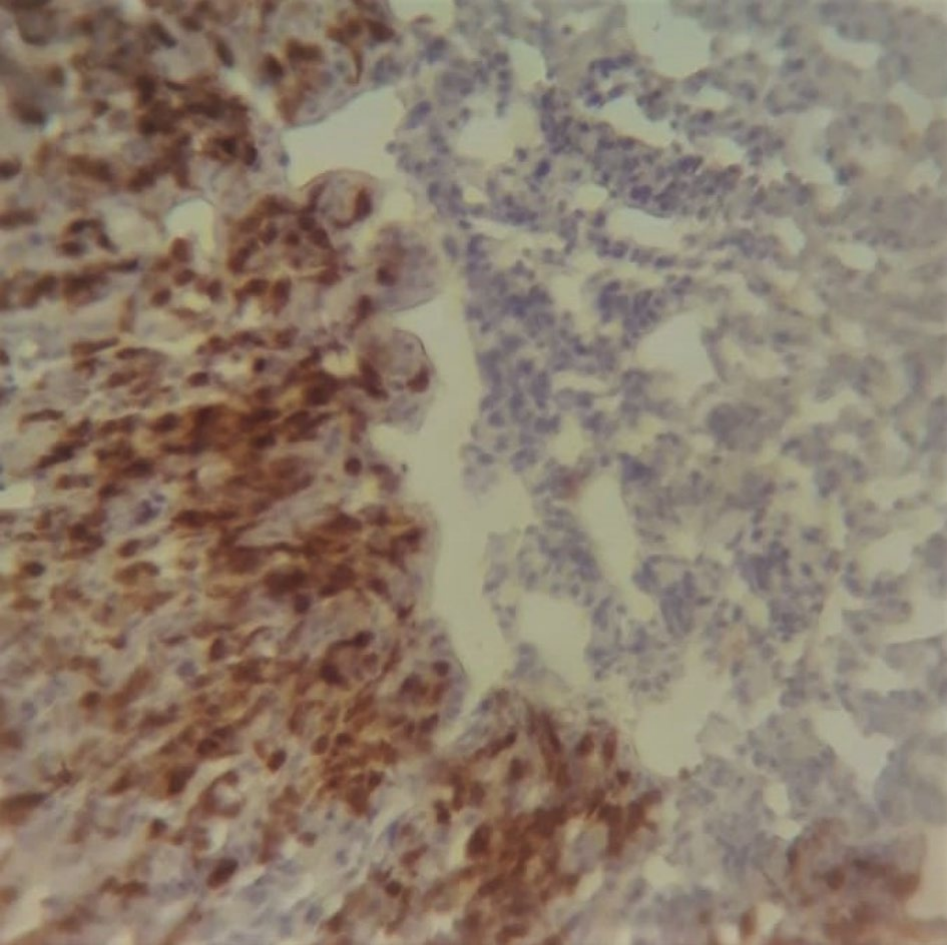
Section of the caecum of broiler infected by *Salmonella* Typhimurium ∆*ttr*A∆*pdu*A showing immunoreactions in macrophages, 7 days post-infection (× 400; Avidin–Biotin Streptavidin Peroxidase, counterstained with Hematoxylin).

On the following day, the slides were washed as previously. Then, droplets of the secondary antibody (Reveal Complement, Abcam©, USA) was added after removing the excess PBS and the humidity chamber was placed in a dark ambient for 30 min. Subsequently, a drop of 3,3′-diaminobenzidine (DAB Chromogen 50 ×, Abcam©, USA) was diluted in 1 mL of substrate (DAB Substrate, Abcam©, USA), which volume is enough for three slides, and added to the tissue sections. One minute later, the slides were submerged in 200 mL dH_2_O for 5 min. After that, they were transferred to a plastic cube containing Harris hematoxylin (Êxodo Científica, Brazil) and were left for 1 min. Posteriorly, the slides were washed for 10 min under running water at low pressure. The slides were submitted to the alcohol-xylene series (70% Alcohol, 90% Alcohol, 100% Alcohol, Xylene I, and Xylene II). In the end, the coverslips were placed on the slides after adding a drop of water-free mounting medium (Entellan^®^, Merck, Brazil). Images from the tissue sections were taken randomly, choosing five random view fields, using an optical microscope (lens 400 ×) (Coleman^®^, model N-120) with a digital camera adapter, for further statistical analysis (Fig. 1).

### Data analysis

The percentage of CD4+ and CD8+ cells and macrophages was calculated using Image-Pro Plus v.4.5.0.29 (MediaCybernetics, USA). They were quantified as percentage values by the immune cell marker positive area/total area. Statistical analysis and graphics were done using the software GraphPad Prism v.8.0.1 for macOS (GraphPad Software, La Jolla California, USA) and data were submitted to Variance Analysis (ANOVA) followed by Bonferroni multiple comparisons, considering a significance level lower than 5% (P ≤ 0.05).

### Ethical statement

The authors declare that all the in vivo experiment was performed in complete accordance with relevant guidelines and regulations. Moreover, the authors declare that the study was carried out in accordance with ARRIVE guidelines (https://arriveguidelines.org).

## Supplementary Information

Supplementary Figures.

## Data Availability

The data supporting this study's findings are available from the corresponding author, upon reasonable request.
