# Supplementary material for: Salmonella enterica serovars in absence of ttrA and pduA genes enhance the cell immune response during chick infections
Source: Sci Rep. 2023 Jan 11;13:595. doi: 10.1038/s41598-023-27741-x (PMC9834210; doi:10.1038/s41598-023-27741-x)
Supplement: Supplementary file 1 — Supplementary Figures. [file 41598_2023_27741_MOESM1_ESM.pdf]

Salmonella enterica serovars in absence of *ttr*A and *pdu*A genes enhance the cell immune response during chick infections

Julia Memrava Cabrera[1], Mauro de Mesquita Souza Saraiva[1,2*], Daniel Farias Marinho do Monte[1], Lucas Bocchini Rodrigues Alves[1], Rosemeri de Oliveira Vasconcelos[1], Oliveiro Caetano de Freitas Neto[3], Angelo Berchieri Junior[1]

[1] Department of Pathology, Theriogenology, and One Health, Sao Paulo State University (FCAV-Unesp), Jaboticabal, SP, Brazil; [2] Department of Veterinary and Animal Sciences, University of Copenhagen, Frederiksberg, Denmark; [3] Department of Preventive Veterinary Medicine, Veterinary School, Federal University of Minas Gerais (UFMG), Belo Horizonte, Brazil.

*Mauro M. S. Saraiva, Department of Pathology, Theriogenology, and One Health, School of Agricultural and Veterinarian Sciences, São Paulo State University (Unesp), 14884-900, Jaboticabal-SP, Brazil, saraiva_ufba@hotmail.com / mauro.saraiva@unesp.br (MMSS);

Figure legends

Supplementary Figure S1. Percentage of the stained area by populations of lymphocytes T CD4$^+$ and CD8$^+$, and macrophages in the caecal tonsils, liver, caecum, and ileum of broiler infected with *Salmonella* Enteritidis or *Salmonella* Enteritidis Δ*ttr*AΔ*pdu*A strains at different days post-infection. Different letters mean a significant statistical difference between challenged (mutant- and wild-type) and no challenged birds, in each of the days post-inoculation (dpi), by two-way ANOVA followed by Bonferroni multiple comparison test at 5% probability.

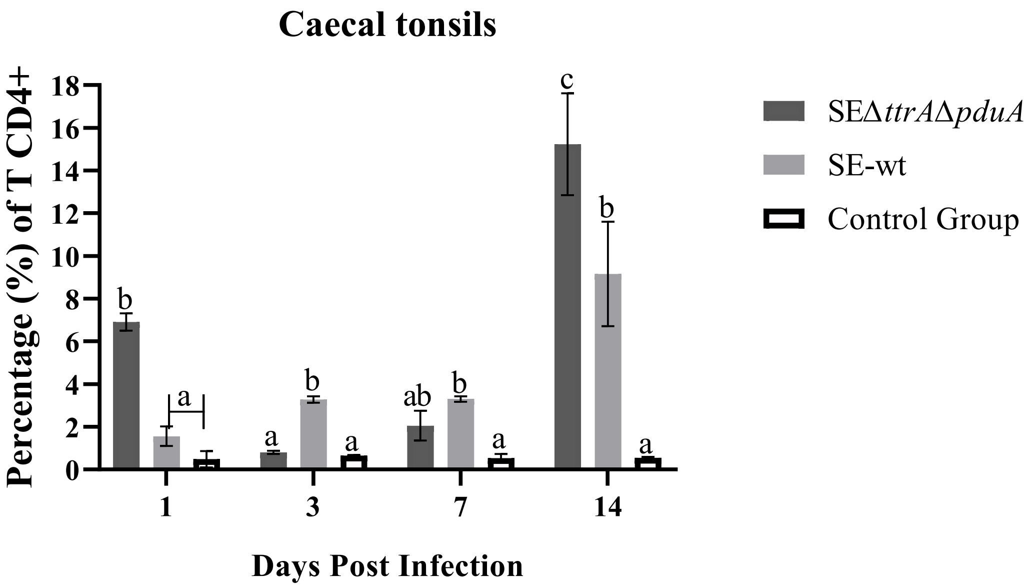
Caecal tonsils
Percentage (%) of T CD4+
18
16
14
12
10
8
6
4
2
0
1
3
7
14
Days Post Infection
SEΔttrAΔpduA
SE-wt
Control Group
a
b
ab
c

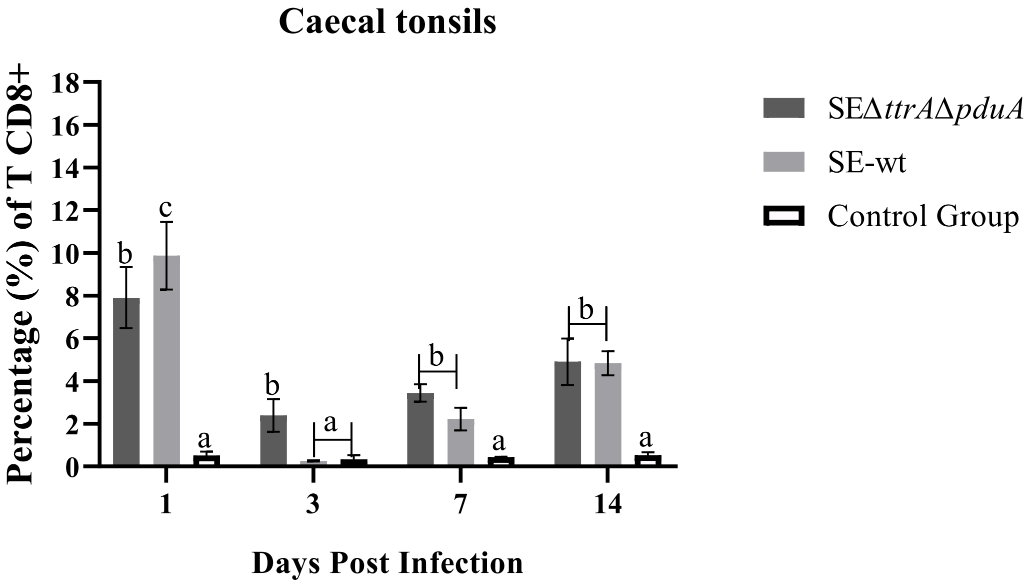
Caecal tonsils
Percentage (%) of T CD8+
0
2
4
6
8
10
12
14
16
18
b
c
a
b
a
b
a
b
a
1
3
7
14
Days Post Infection
SEΔ*ttrA*Δ*pduA*
SE-wt
Control Group

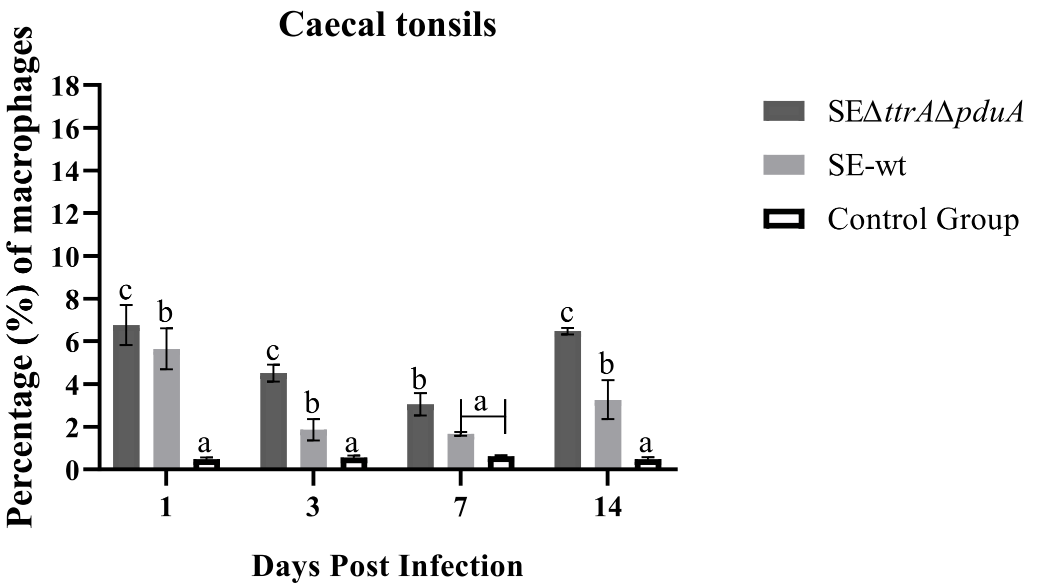

Caecal tonsils
Percentage (%) of macrophages
18
16
14
12
10
8
6
4
2
0
c
b
a
c
b
a
b
a
c
b
a
1
3
7
14
Days Post Infection
SEΔ*ttrA*Δ*pduA*
SE-wt
Control Group

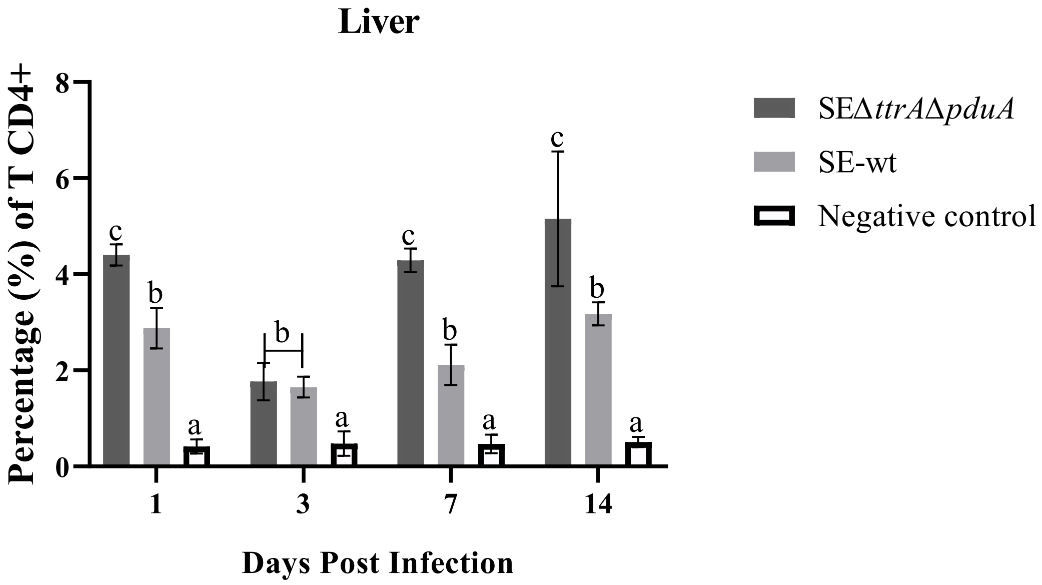
Liver
Percentage (%) of T CD4+
8
6
4
2
0
c
b
a
b
a
c
b
a
c
b
a
1
3
7
14
Days Post Infection
SEΔttrAΔpduA
SE-wt
Negative control

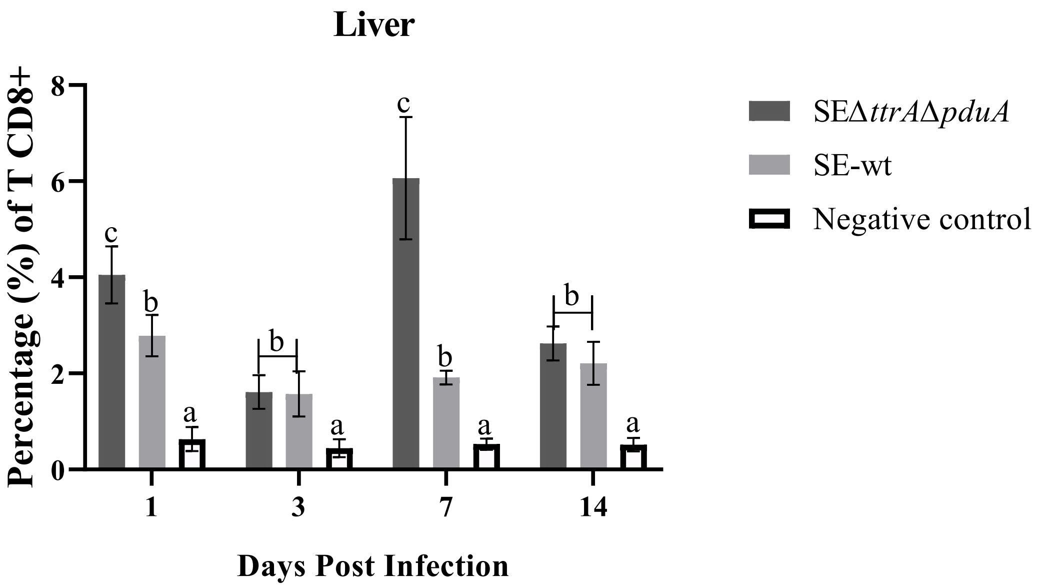

Liver
Percentage (%) of T CD8+
8
6
4
2
0
c
b
a
b
a
c
b
a
b
a
1
3
7
14
Days Post Infection
SEΔttrAΔpduA
SE-wt
Negative control

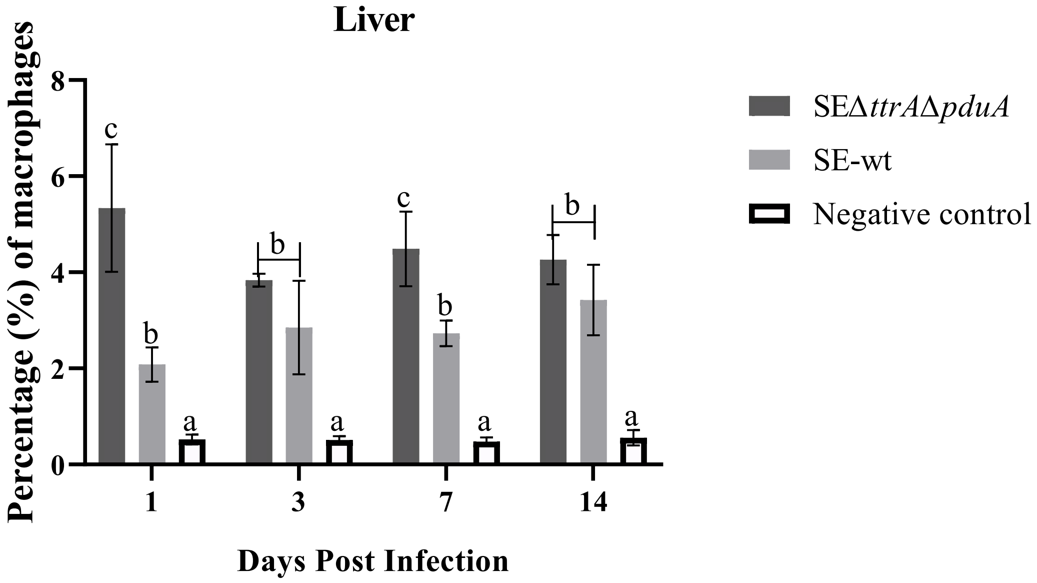
Liver
Percentage (%) of macrophages
0
2
4
6
8
c
b
a
b
a
c
b
a
b
a
1
3
7
14
Days Post Infection
SEΔttrAΔpduA
SE-wt
Negative control

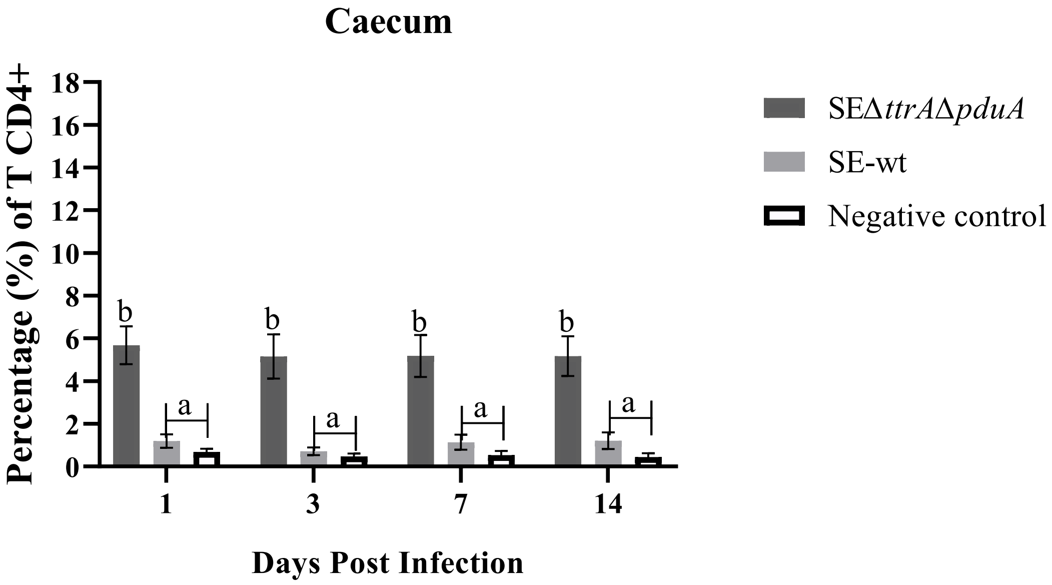

Caecum
Percentage (%) of T CD4+
18
16
14
12
10
8
6
4
2
0
b
a
1
3
7
14
Days Post Infection
SEΔttrAΔpduA
SE-wt
Negative control

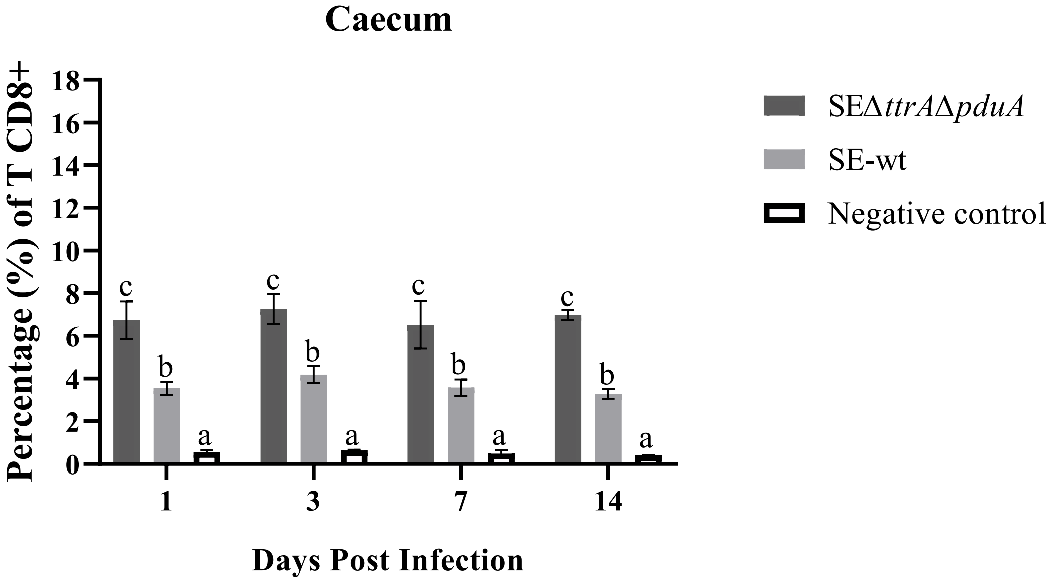
Caecum
Percentage (%) of T CD8+
18
16
14
12
10
8
6
4
2
0
c
b
a
1
3
7
14
Days Post Infection
SEΔ*ttrA*Δ*pduA*
SE-wt
Negative control

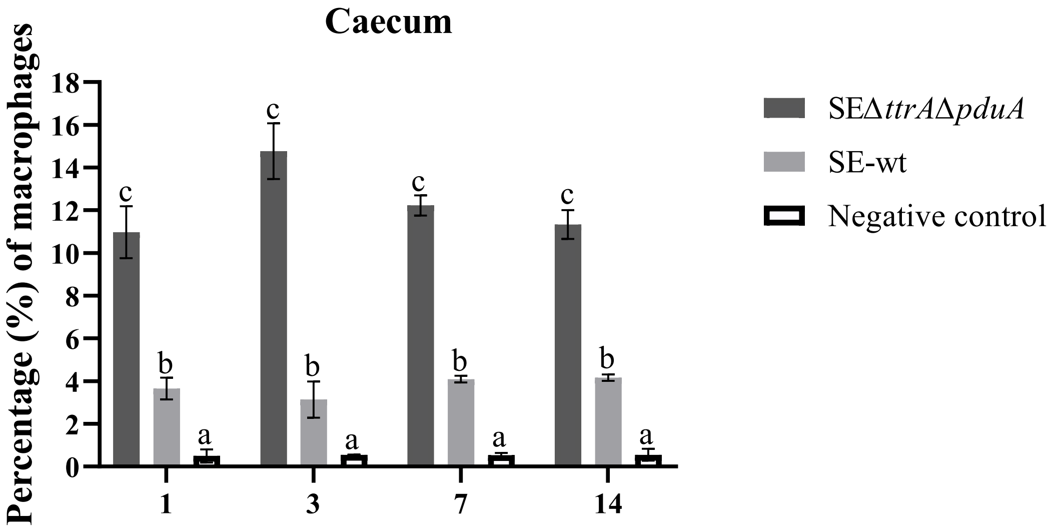
Caecum
Percentage (%) of macrophages
0
2
4
6
8
10
12
14
16
18
c
b
a
1
3
7
14
Days Post Infection
SEΔ*ttrA*Δ*pduA*
SE-wt
Negative control

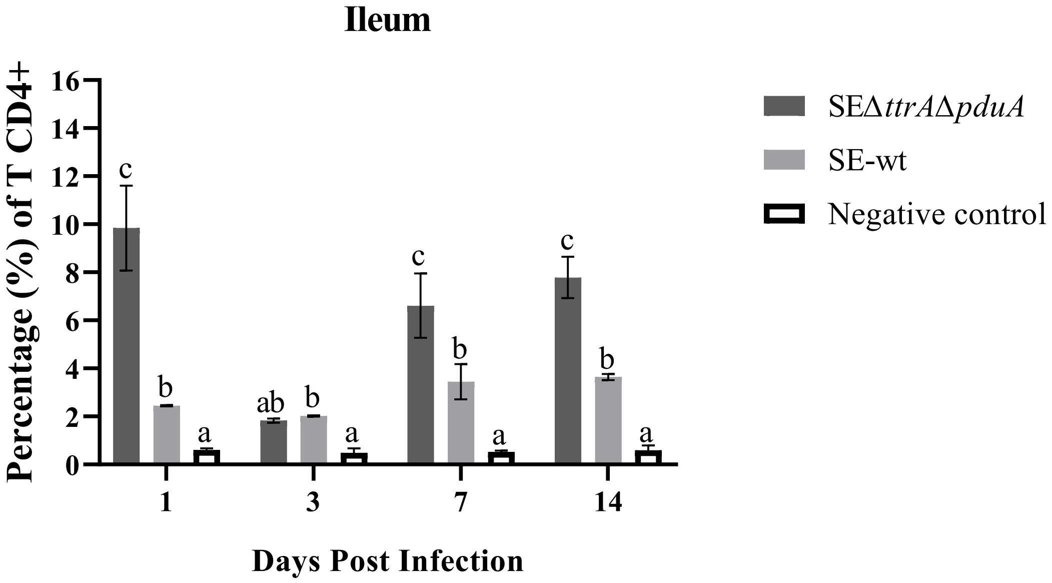

Ileum
Percentage (%) of T CD4+
16
14
12
10
8
6
4
2
0
c
b
a
ab
b
a
c
b
a
c
b
a
1
3
7
14
Days Post Infection
SEΔttrAΔpduA
SE-wt
Negative control

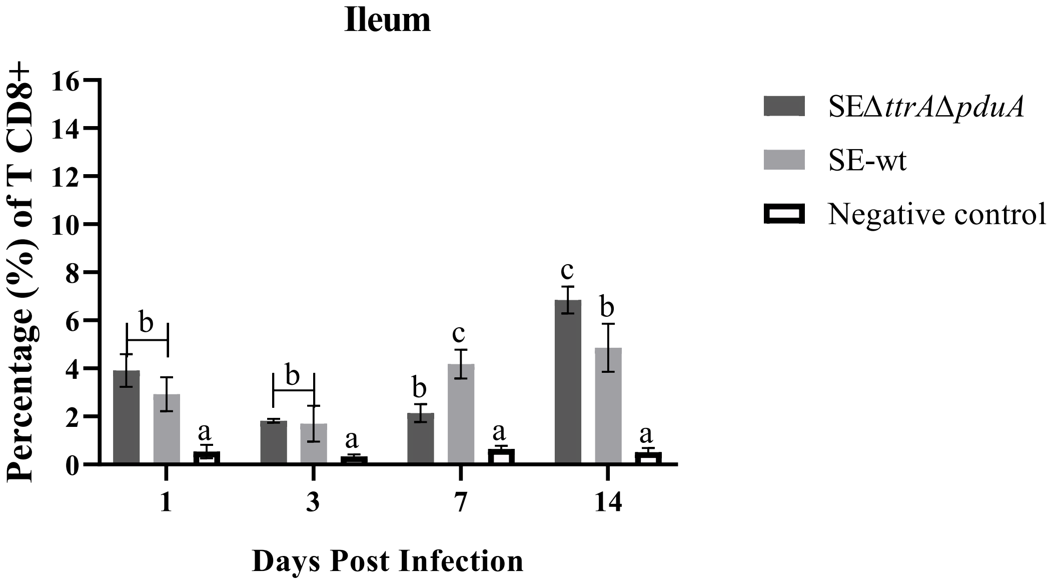
Ileum
Percentage (%) of T CD8+
16
14
12
10
8
6
4
2
0
b
b
a
a
b
c
a
c
b
a
1
3
7
14
Days Post Infection
SEΔttrAΔpduA
SE-wt
Negative control

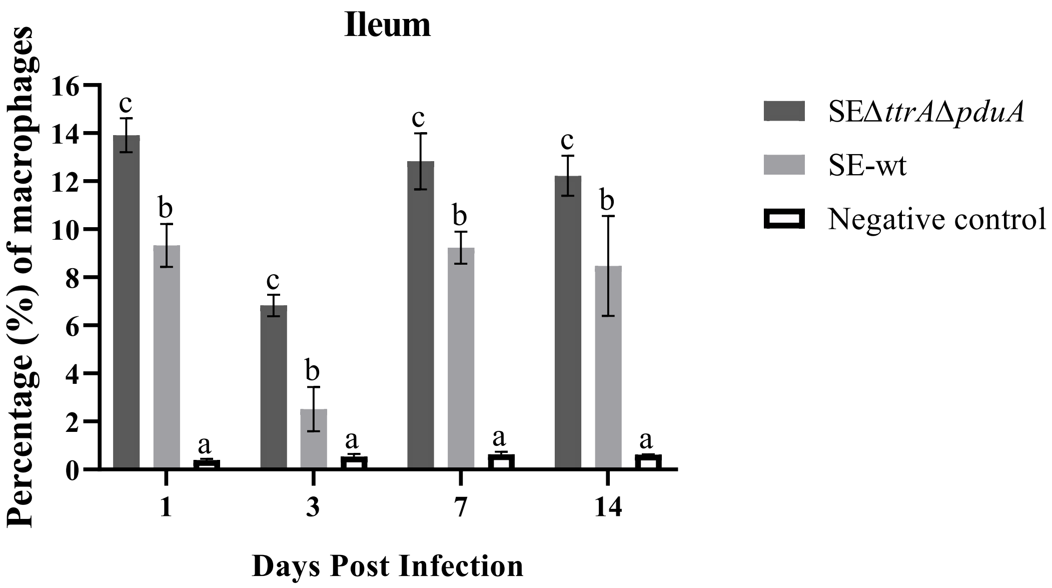

Ileum
Percentage (%) of macrophages
16
14
12
10
8
6
4
2
0
c
b
a
1
3
7
14
Days Post Infection
SEΔttrAΔpduA
SE-wt
Negative control

Supplementary Figure S2. Percentage of the stained area by populations of lymphocytes T $CD4^+$ and $CD8^+$, and macrophages in the caecal tonsils, liver, caecum, and ileum of semi-heavy laying hens infected with *Salmonella* Enteritidis or *Salmonella* Enteritidis Δ*ttr*AΔ*pdu*A strains at different days post-infection. Different letters mean a significant statistical difference between challenged (mutant- and wild-type) and no challenged birds, in each of the days post-inoculation (dpi), by two-way ANOVA followed by Bonferroni multiple comparison test at 5% probability.

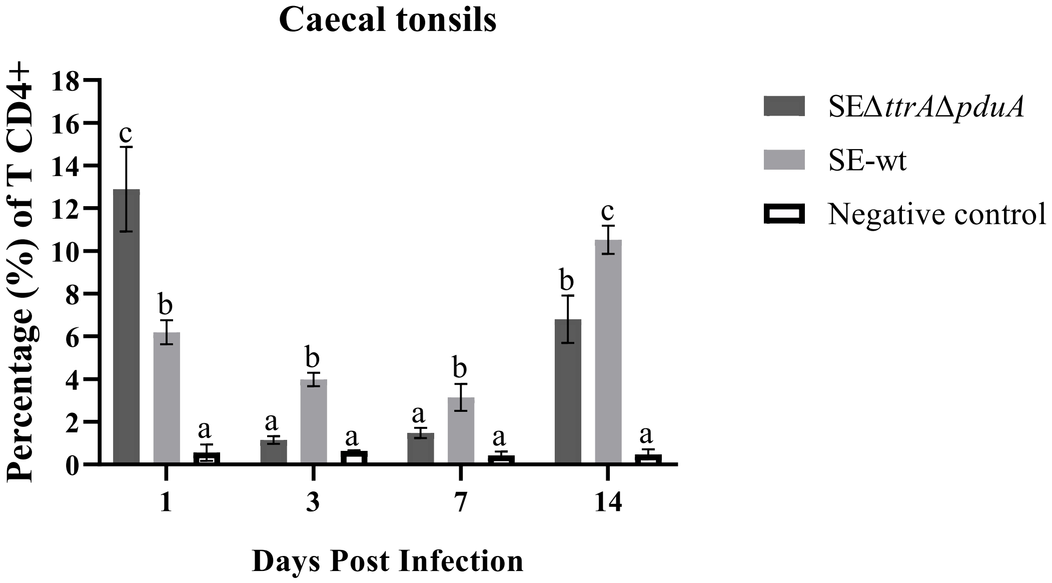
Caecal tonsils
Percentage (%) of T CD4+
18
16
14
12
10
8
6
4
2
0
c
b
a
a
b
a
a
b
a
b
c
a
1
3
7
14
Days Post Infection
SEΔttrAΔpduA
SE-wt
Negative control

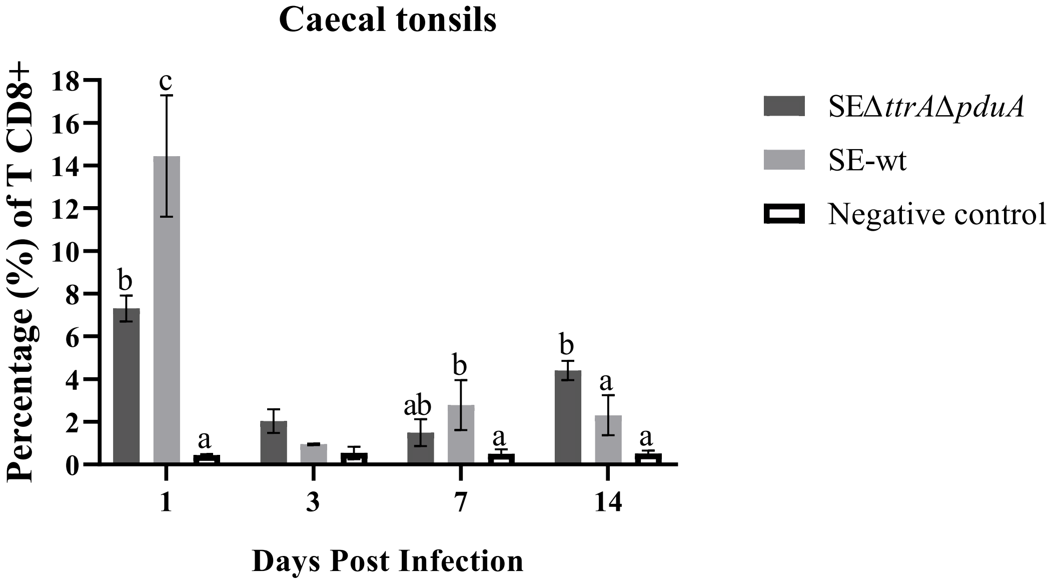

Caecal tonsils
Percentage (%) of T CD8+
18
16
14
12
10
8
6
4
2
0
c
b
a
ab
b
a
b
a
a
1
3
7
14
Days Post Infection
SEΔ*ttrA*Δ*pduA*
SE-wt
Negative control

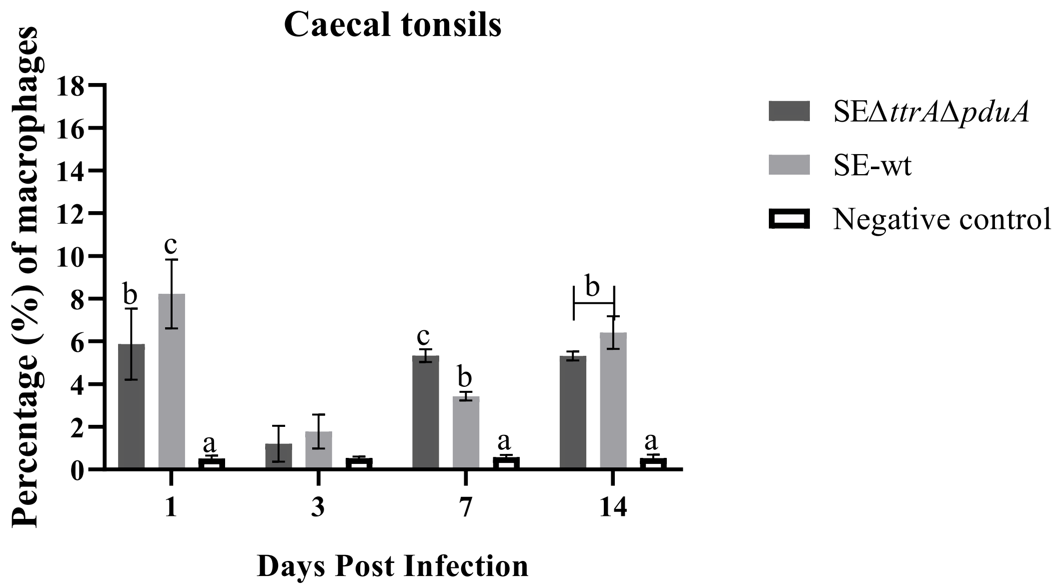
Caecal tonsils
Percentage (%) of macrophages
0
2
4
6
8
10
12
14
16
18
b
c
a
c
b
a
b
a
1
3
7
14
Days Post Infection
SEΔttrAΔpduA
SE-wt
Negative control

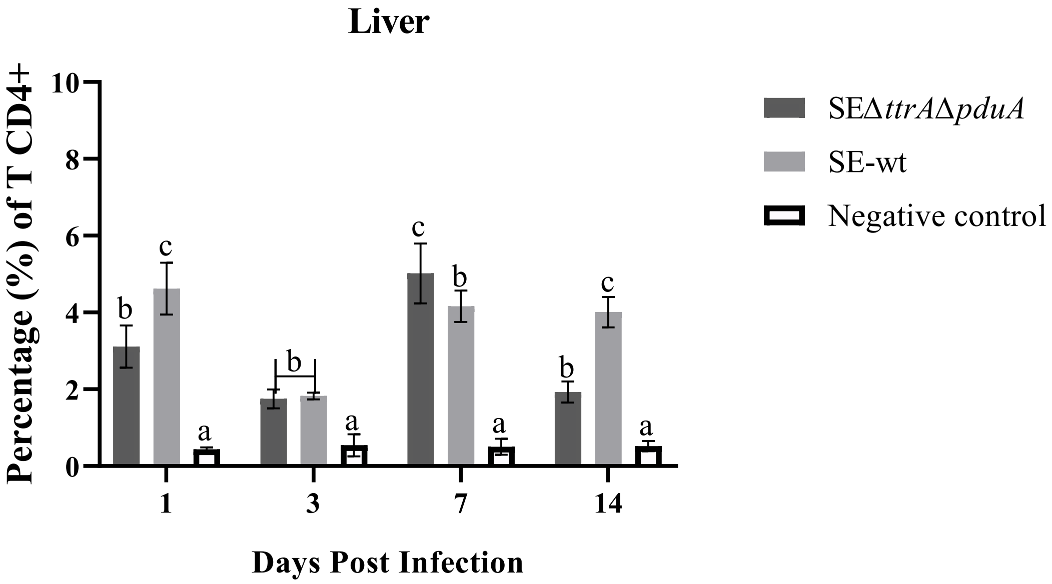
Liver
Percentage (%) of T CD4+
10
8
6
4
2
0
b
c
a
b
a
c
b
a
b
c
a
1
3
7
14
Days Post Infection
SEΔttrAΔpduA
SE-wt
Negative control

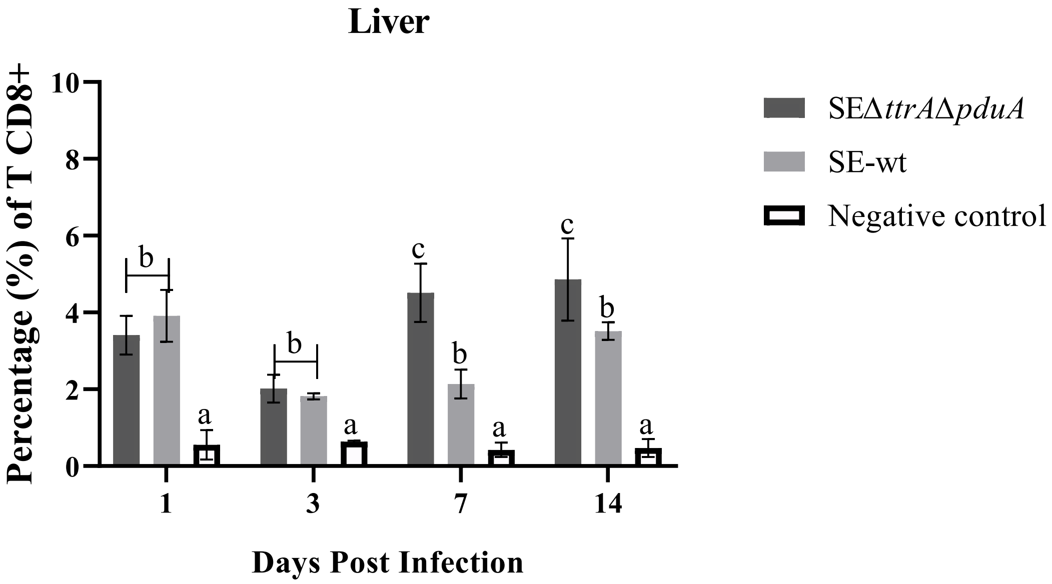

Liver
Percentage (%) of T CD8+
0
2
4
6
8
10
b
b
a
a
c
b
a
c
b
a
1
3
7
14
Days Post Infection
SEΔttrAΔpduA
SE-wt
Negative control

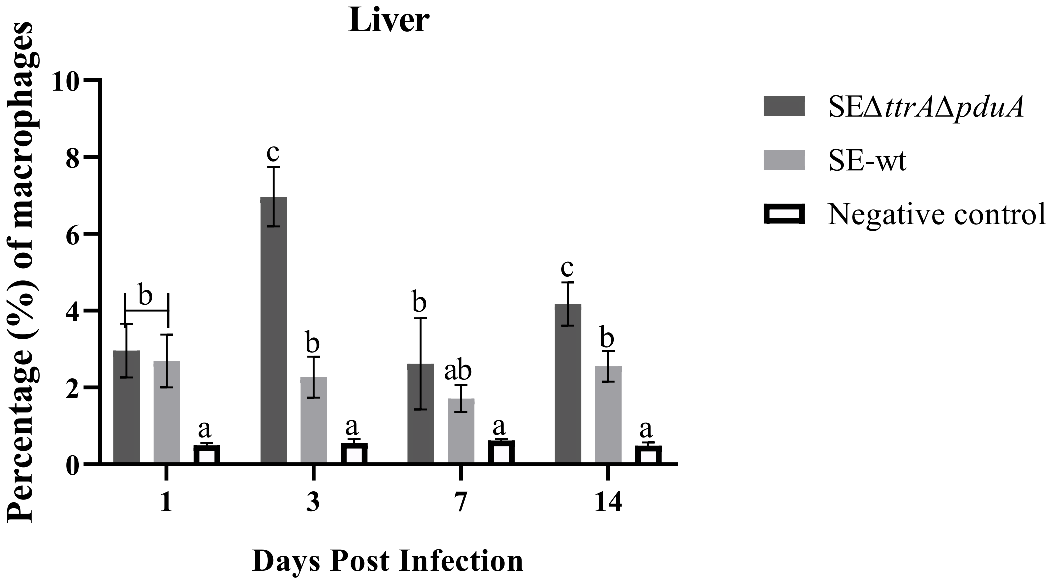
Liver
Percentage (%) of macrophages
10
8
6
4
2
0
c
b
c
b
b
ab
b
a
a
a
a
1
3
7
14
Days Post Infection
SEΔttrAΔpduA
SE-wt
Negative control

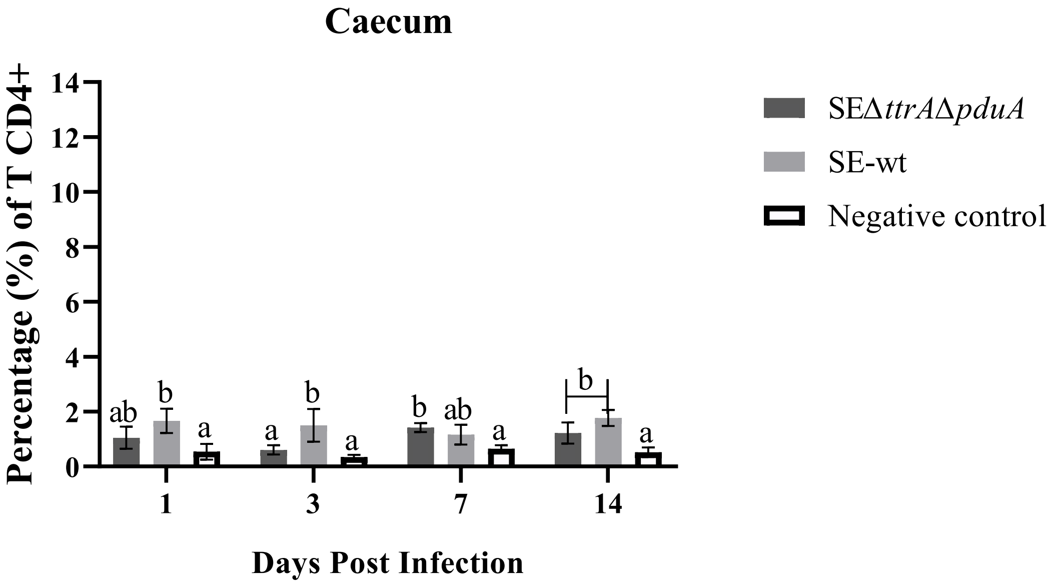
Caecum
Percentage (%) of T CD4+
14
12
10
8
6
4
2
0
ab
b
a
a
b
a
b
ab
a
b
a
1
3
7
14
Days Post Infection
SEΔttrAΔpduA
SE-wt
Negative control

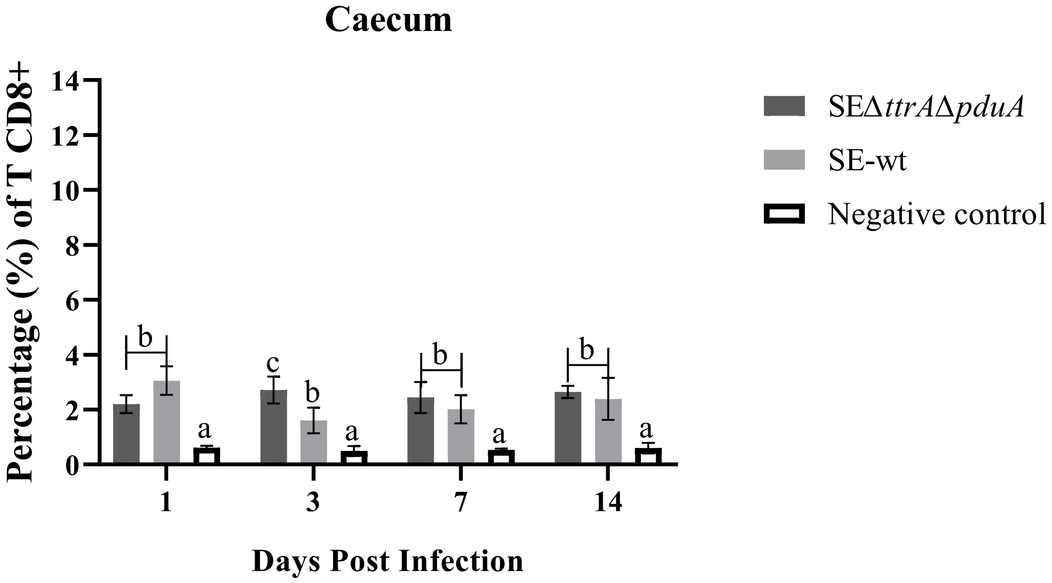
Caecum
Percentage (%) of T CD8+
14
12
10
8
6
4
2
0
b
c
b
a
a
b
a
b
a
1
3
7
14
Days Post Infection
SEΔttrAΔpduA
SE-wt
Negative control

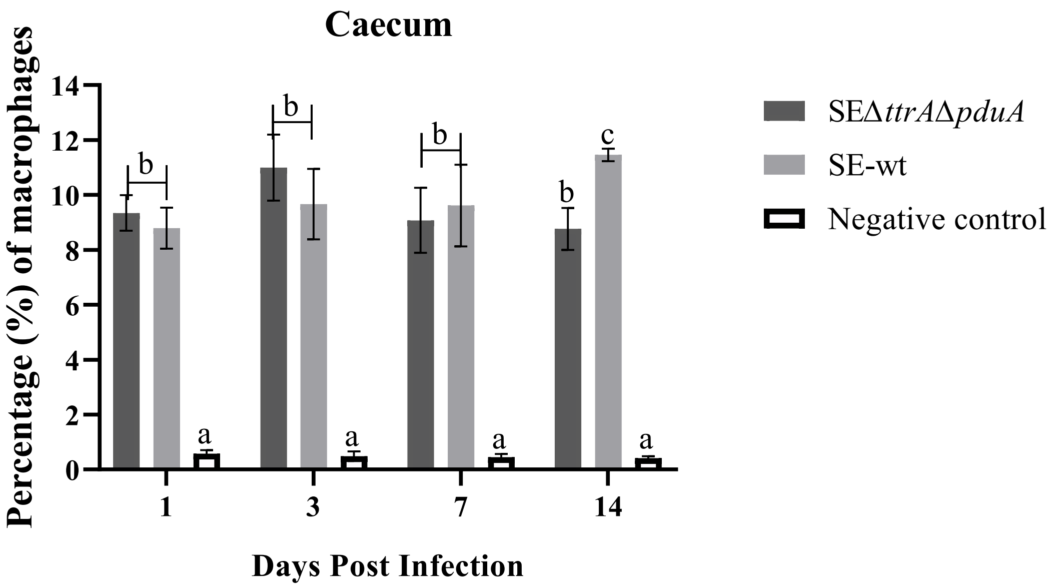

Caecum
Percentage (%) of macrophages
14
12
10
8
6
4
2
0
b
b
b
b
c
a
a
a
a
1
3
7
14
Days Post Infection
SEΔttrAΔpduA
SE-wt
Negative control

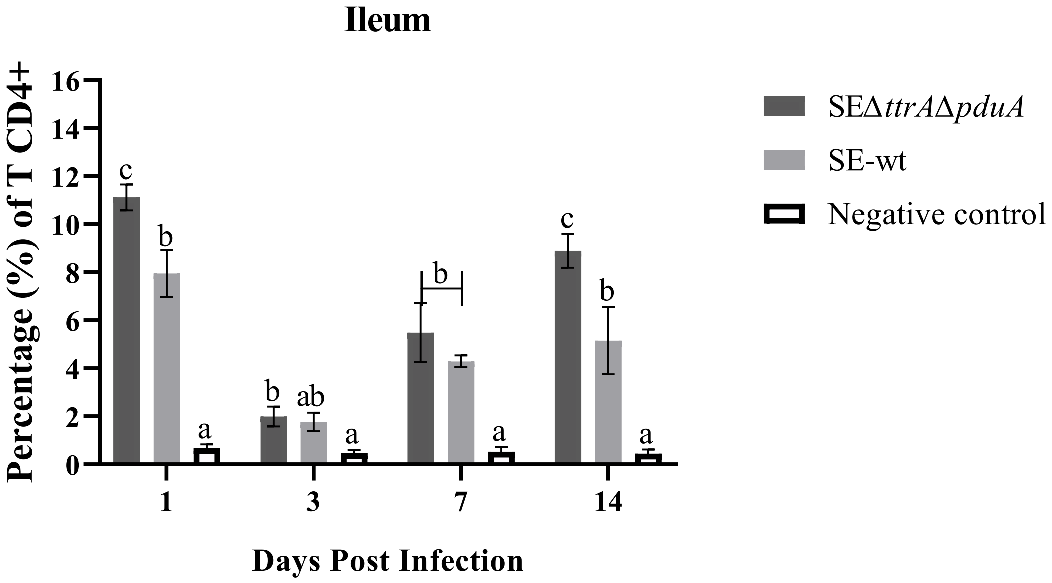
Ileum
Percentage (%) of T CD4+
16
14
12
10
8
6
4
2
0
c
b
a
b
ab
a
b
a
c
b
a
1
3
7
14
Days Post Infection
SEΔttrAΔpduA
SE-wt
Negative control

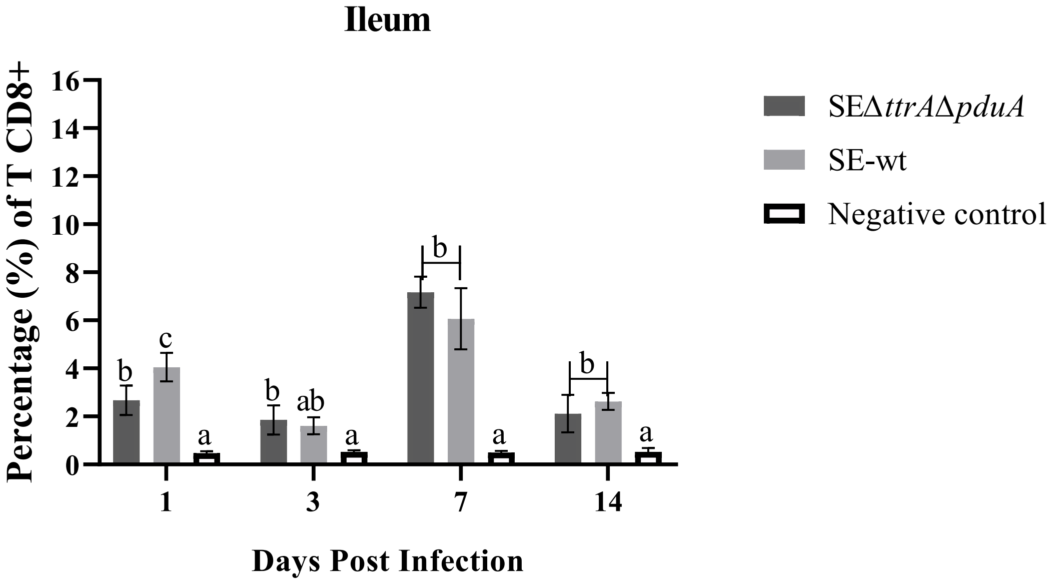

Ileum
Percentage (%) of T CD8+
16
14
12
10
8
6
4
2
0
b
c
a
b
ab
a
b
a
b
a
1
3
7
14
Days Post Infection
SEΔttrAΔpduA
SE-wt
Negative control

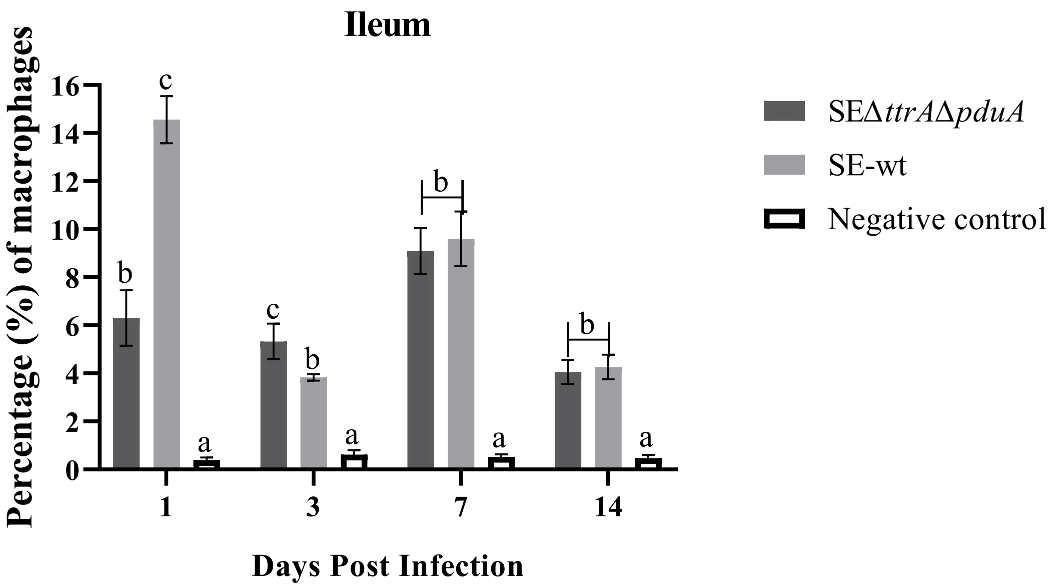

Ileum
Percentage (%) of macrophages
16
14
12
10
8
6
4
2
0
c
b
a
c
b
a
b
a
b
a
1
3
7
14
Days Post Infection
SEΔ*ttrA*Δ*pduA*
SE-wt
Negative control

Supplementary Figure S3. Percentage of the stained area by populations of lymphocytes T $CD4^+$ and $CD8^+$, and macrophages in the caecal tonsils, liver, caecum, and ileum of light laying hens infected with *Salmonella* Enteritidis or *Salmonella* Enteritidis Δ*ttr*AΔ*pdu*A strains at different days post-infection. Different letters mean a significant statistical difference between challenged (mutant- and wild-type) and no challenged birds, in each of the days post-inoculation (dpi), by two-way ANOVA followed by Bonferroni multiple comparison test at 5% probability.

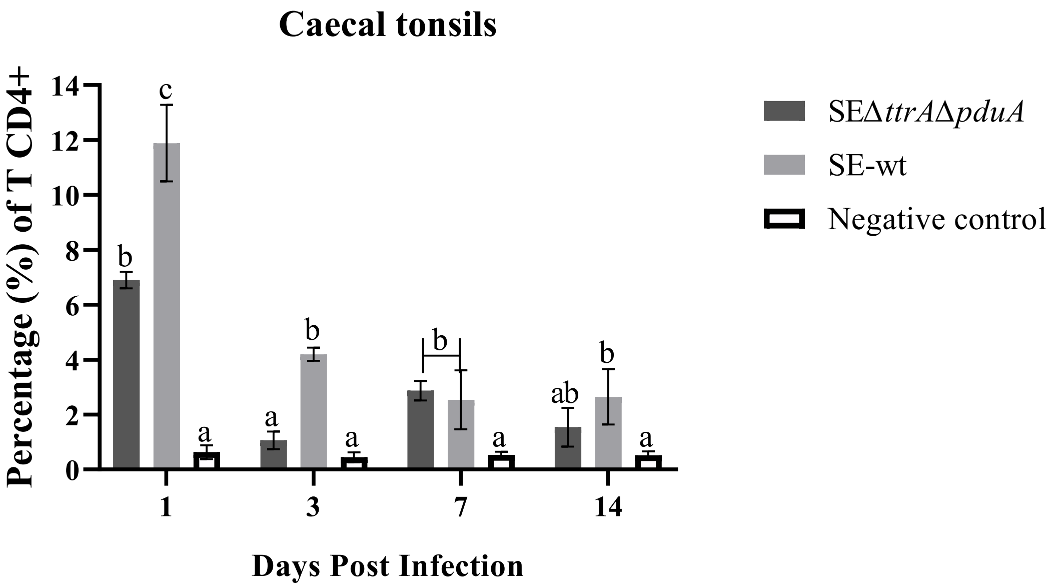
Caecal tonsils
Percentage (%) of T CD4+
Days Post Infection
SEΔttrAΔpduA
SE-wt
Negative control
0
2
4
6
8
10
12
14
1
3
7
14
b
c
a
a
b
a
b
a
ab
b
a

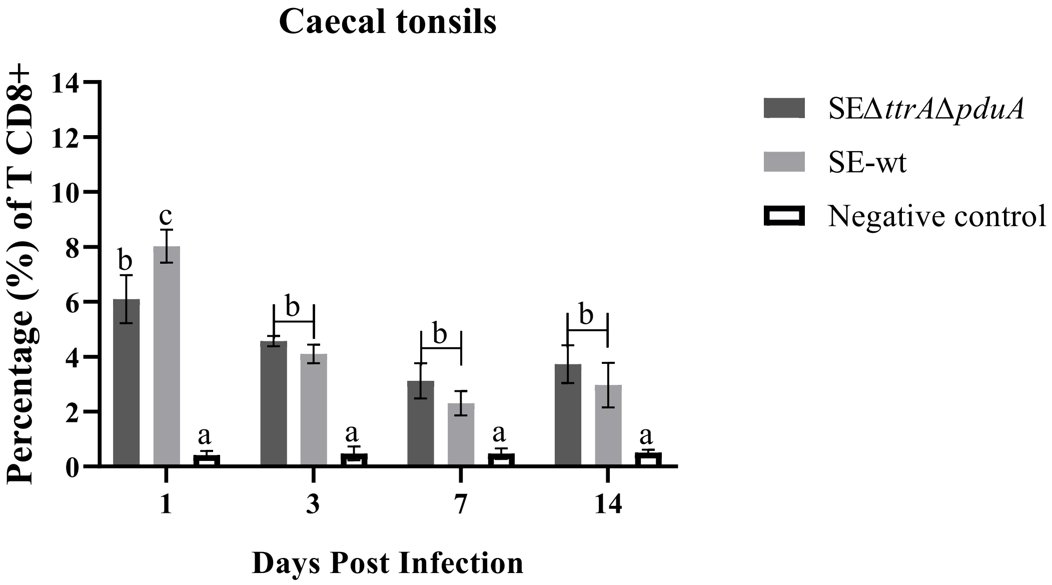
Caecal tonsils
Percentage (%) of T CD8+
0
2
4
6
8
10
12
14
b
c
a
b
a
b
a
b
a
1
3
7
14
Days Post Infection
SEΔ*ttrA*Δ*pduA*
SE-wt
Negative control

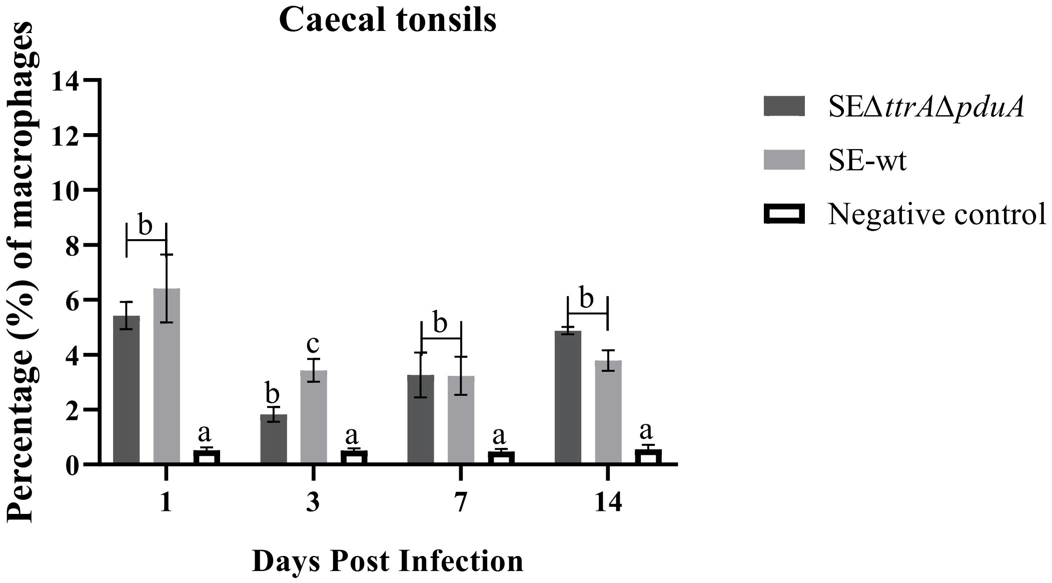

Caecal tonsils
Percentage (%) of macrophages
14
12
10
8
6
4
2
0
b
b
c
b
b
b
a
a
a
a
1
3
7
14
Days Post Infection
SEΔttrAΔpduA
SE-wt
Negative control

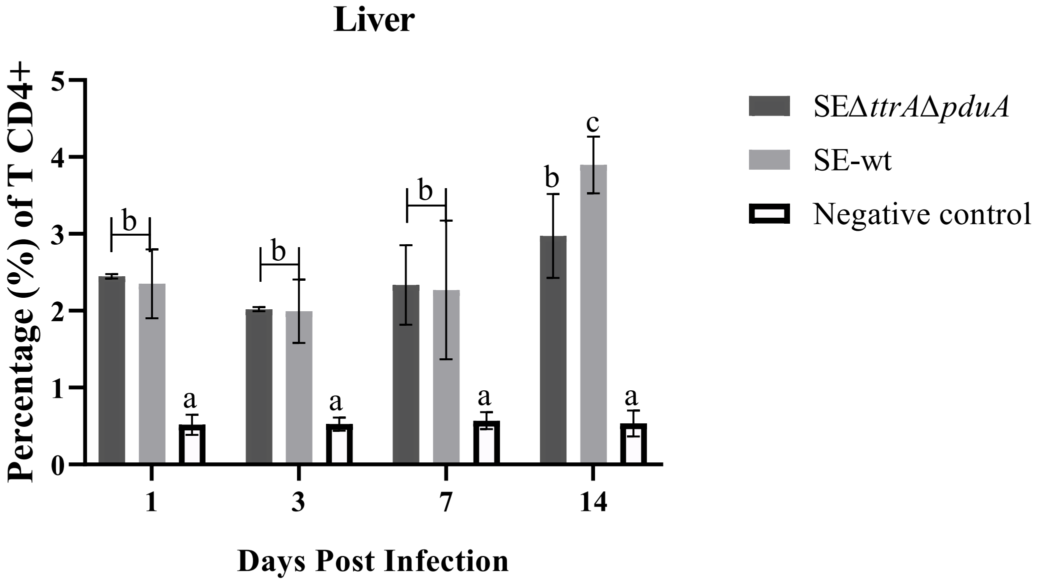
Liver
Percentage (%) of T CD4+
5
4
3
2
1
0
b
b
b
b
c
a
a
a
a
1
3
7
14
Days Post Infection
SEΔttrAΔpduA
SE-wt
Negative control

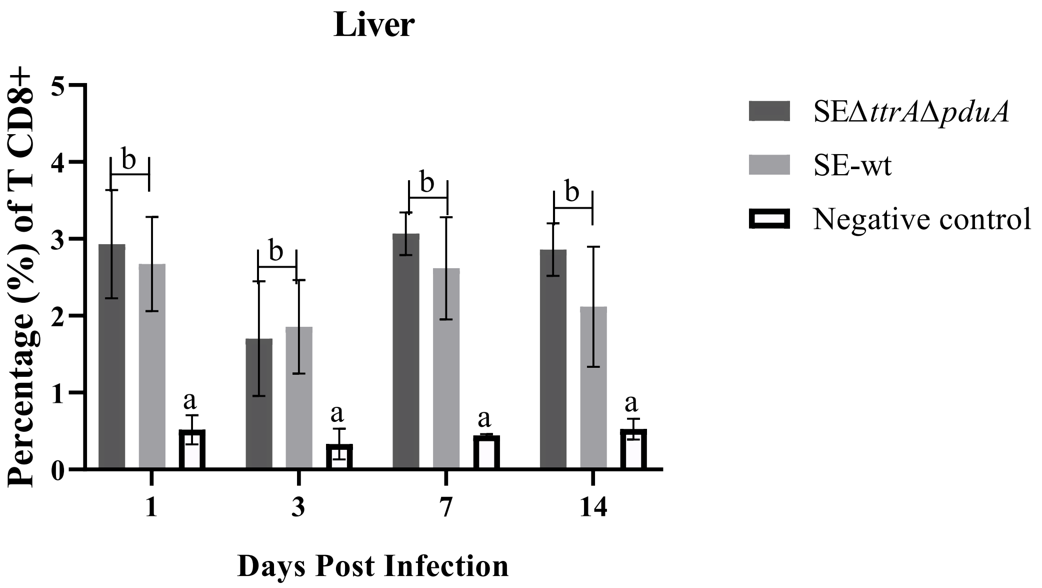
Liver
Percentage (%) of T CD8+
0
1
2
3
4
5
b
a
1
3
7
14
Days Post Infection
SEΔttrAΔpduA
SE-wt
Negative control

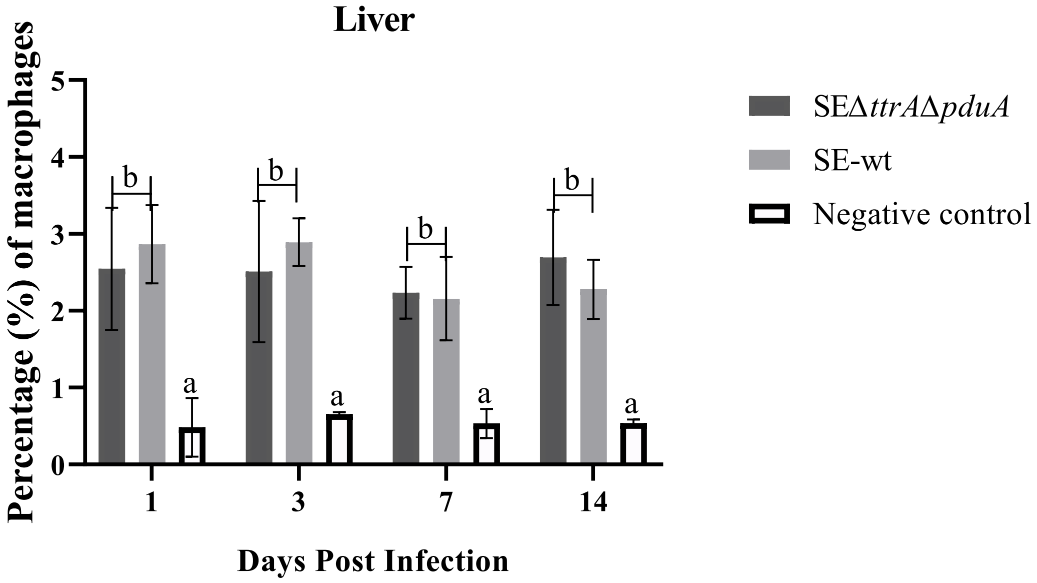
Liver
Percentage (%) of macrophages
5
4
3
2
1
0
b
b
b
b
a
a
a
a
1
3
7
14
Days Post Infection
SEΔttrAΔpduA
SE-wt
Negative control

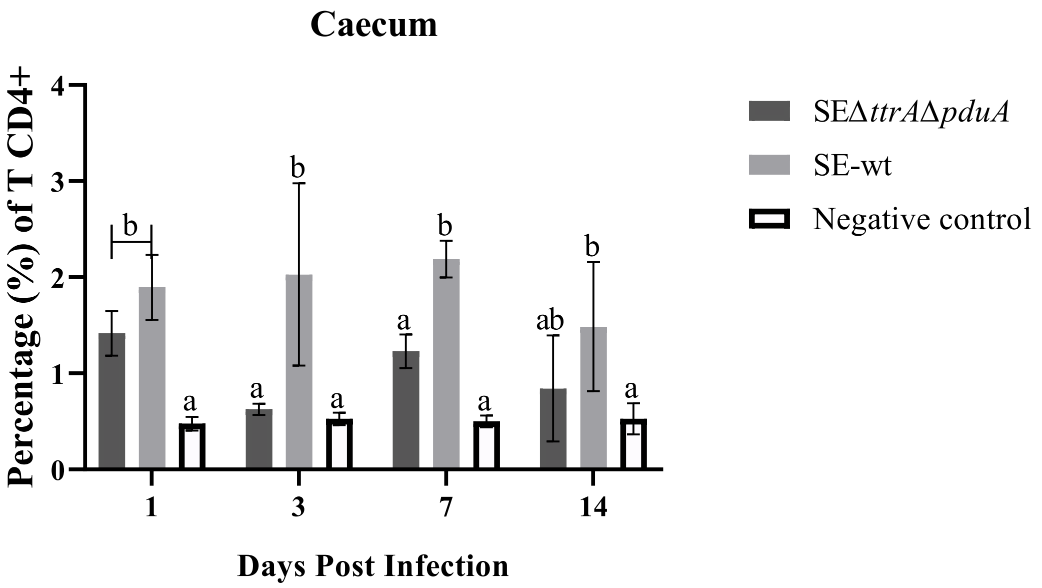

Caecum
Percentage (%) of T CD4+
4
3
2
1
0
b
a
b
a
a
b
a
a
ab
b
a
1
3
7
14
Days Post Infection
SEΔ*ttrA*Δ*pduA*
SE-wt
Negative control

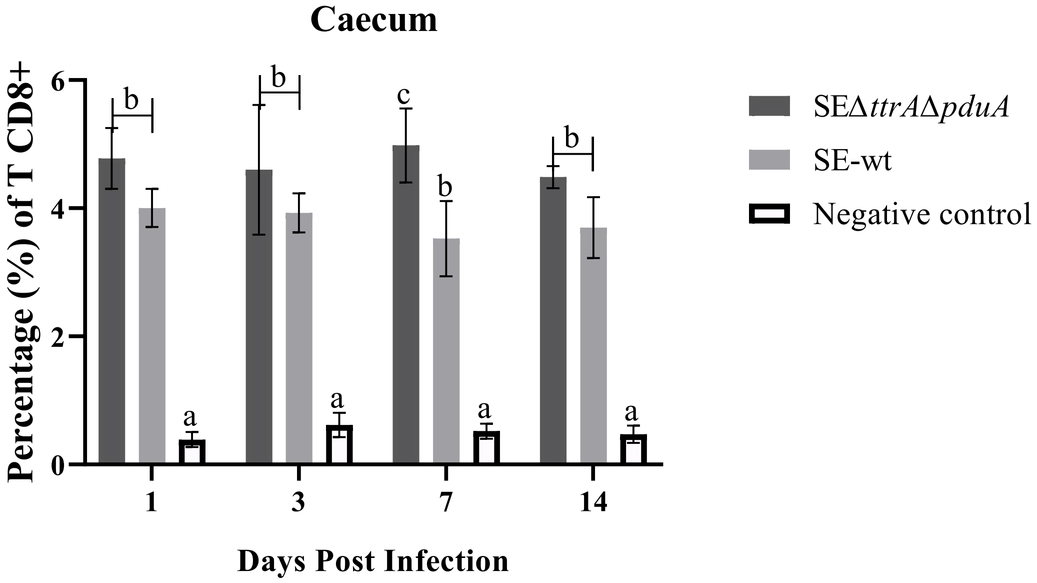
Caecum
Percentage (%) of T CD8+
6
4
2
0
b
b
c
b
b
a
a
a
a
1
3
7
14
Days Post Infection
SEΔttrAΔpduA
SE-wt
Negative control

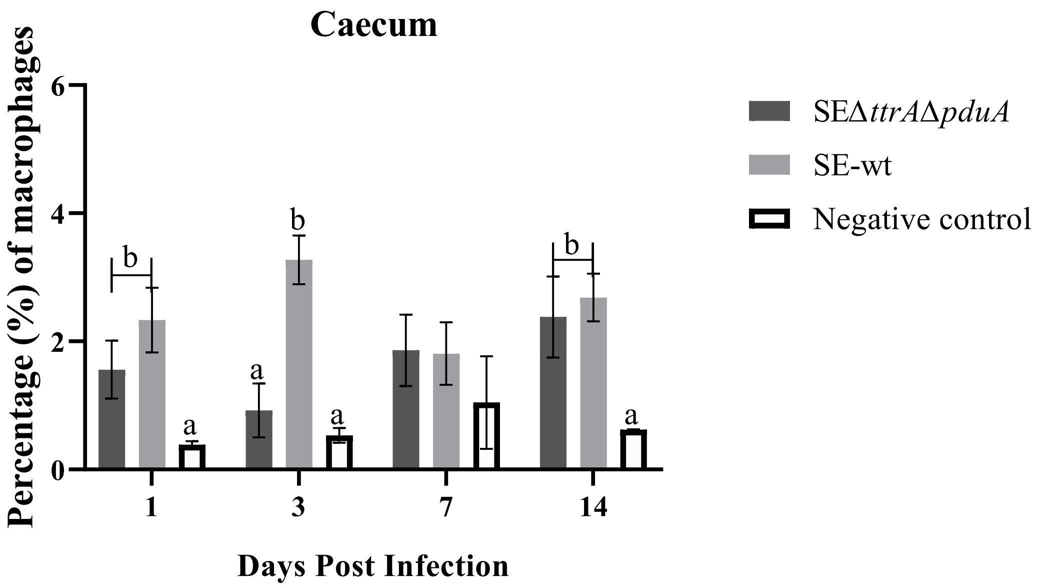
Caecum
Percentage (%) of macrophages
6
4
2
0
b
b
b
a
a
a
a
1
3
7
14
Days Post Infection
SEΔttrAΔpduA
SE-wt
Negative control

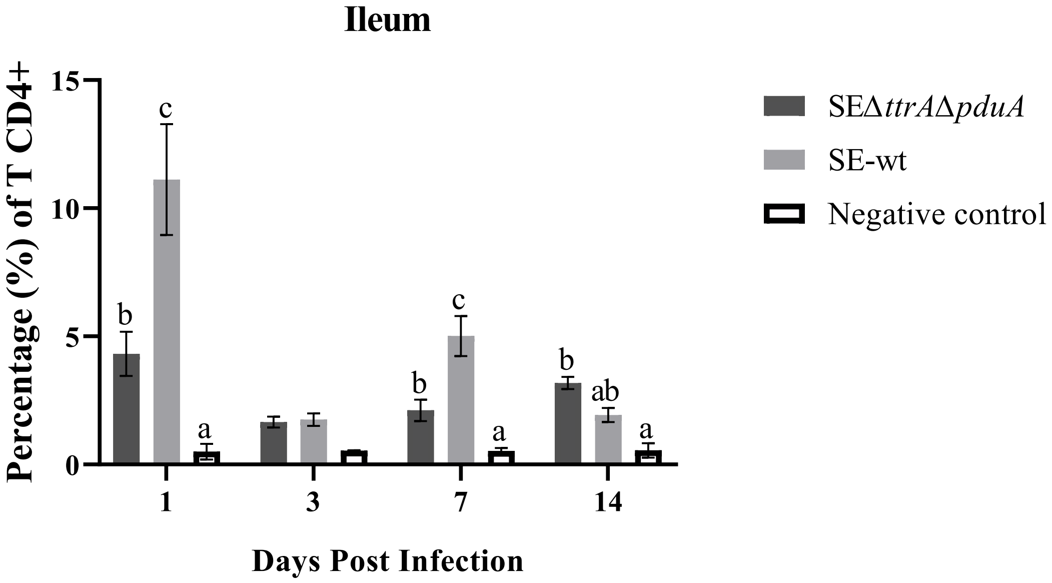

Ileum
Percentage (%) of T CD4+
15
10
5
0
c
b
a
c
b
a
b
ab
a
1
3
7
14
Days Post Infection
SEΔttrAΔpduA
SE-wt
Negative control

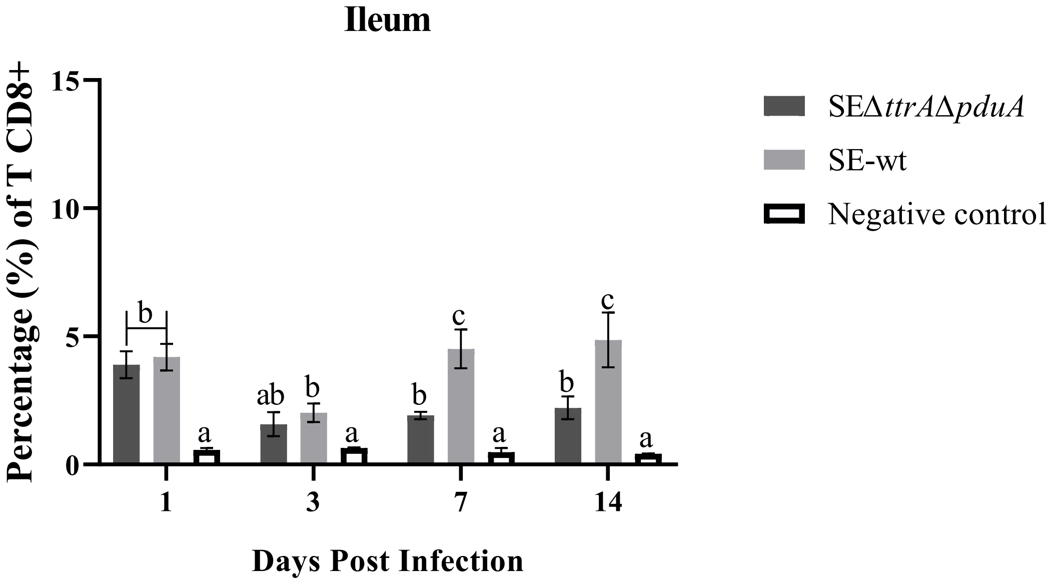

Ileum
Percentage (%) of T CD8+
15
10
5
0
b
ab
b
c
b
c
a
a
a
a
1
3
7
14
Days Post Infection
SEΔ*ttrA*Δ*pduA*
SE-wt
Negative control

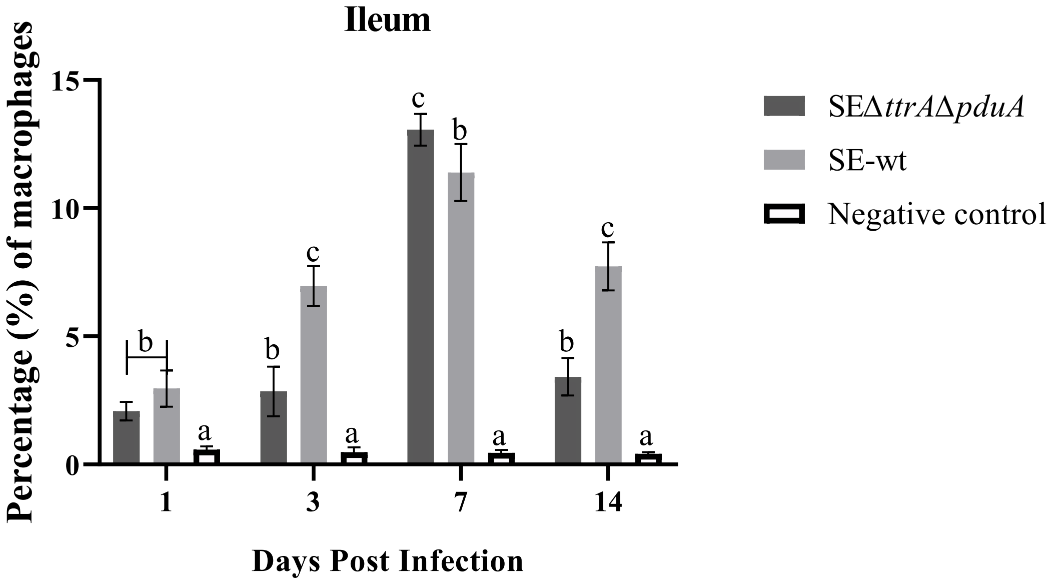

Ileum
Percentage (%) of macrophages
15
10
5
0
c
b
c
b
b
c
b
a
a
a
a
1
3
7
14
Days Post Infection
SEΔttrAΔpduA
SE-wt
Negative control

Supplementary Figure S4. Percentage of the stained area by populations of lymphocytes T $CD4^+$ and $CD8^+$, and macrophages in the caecal tonsils, liver, caecum, and ileum of broiler infected with *Salmonella* Typhimurium or *Salmonella* Typhimurium Δ*ttr*AΔ*pdu*A strains at different days post-infection. Different letters mean a significant statistical difference between challenged (mutant- and wild-type) and no challenged birds, in each of the days post-inoculation (dpi), by two-way ANOVA followed by Bonferroni multiple comparison test at 5% probability.

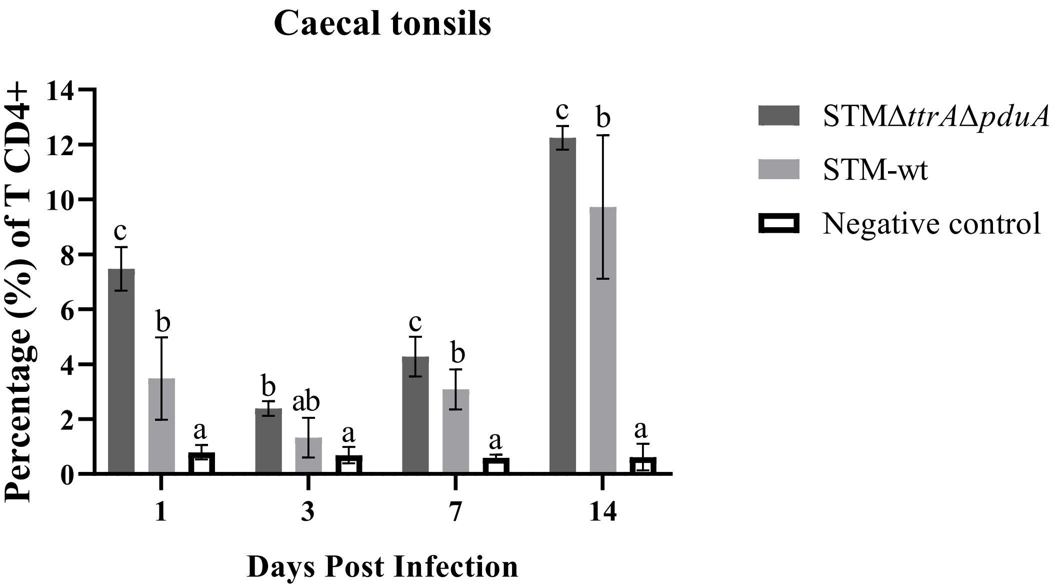

Caecal tonsils
Percentage (%) of T CD4+
0
2
4
6
8
10
12
14
c
b
a
b
ab
a
c
b
a
c
b
a
1
3
7
14
Days Post Infection
STMΔ*ttrA*Δ*pduA*
STM-wt
Negative control

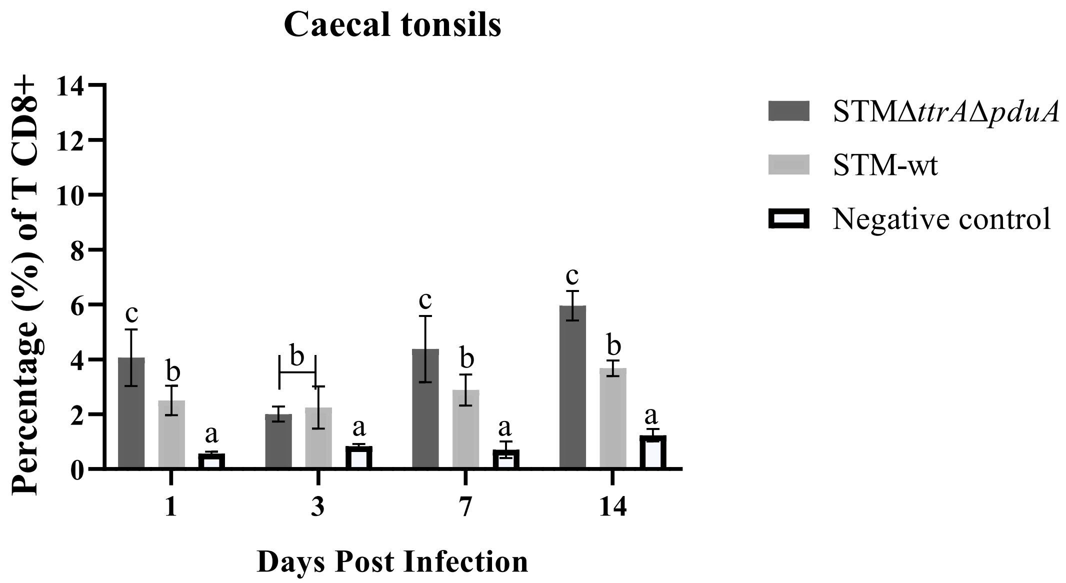

Caecal tonsils
Percentage (%) of T CD8+
14
12
10
8
6
4
2
0
c
b
a
b
a
c
b
a
c
b
a
1
3
7
14
Days Post Infection
STMΔttrAΔpduA
STM-wt
Negative control

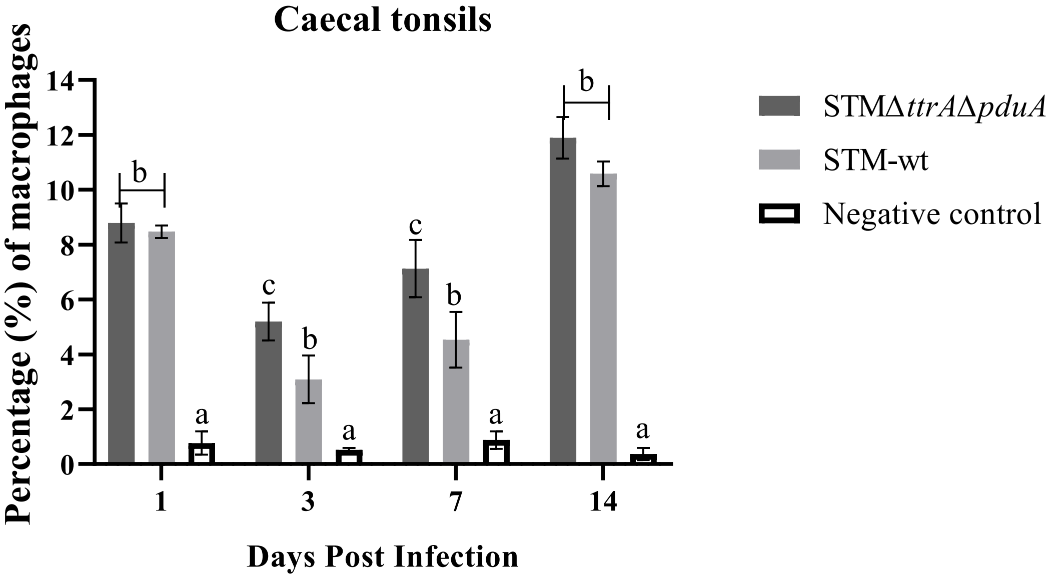

Caecal tonsils
Percentage (%) of macrophages
14
12
10
8
6
4
2
0
b
c
b
a
a
c
b
a
b
a
1
3
7
14
Days Post Infection
STMΔttrAΔpduA
STM-wt
Negative control

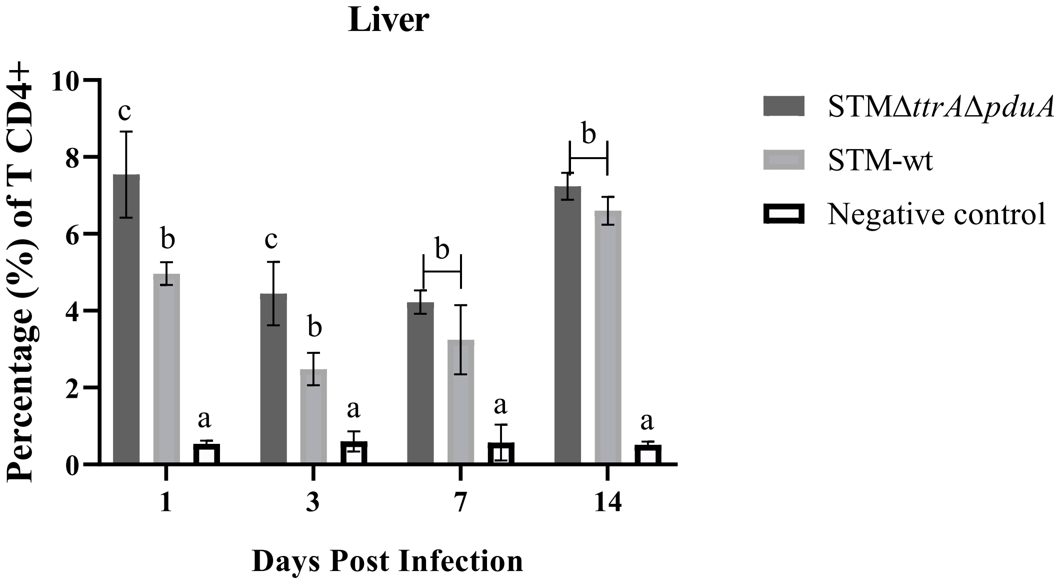
Liver
Percentage (%) of T CD4+
10
8
6
4
2
0
c
b
a
c
b
a
b
a
b
a
1
3
7
14
Days Post Infection
STMΔttrAΔpduA
STM-wt
Negative control

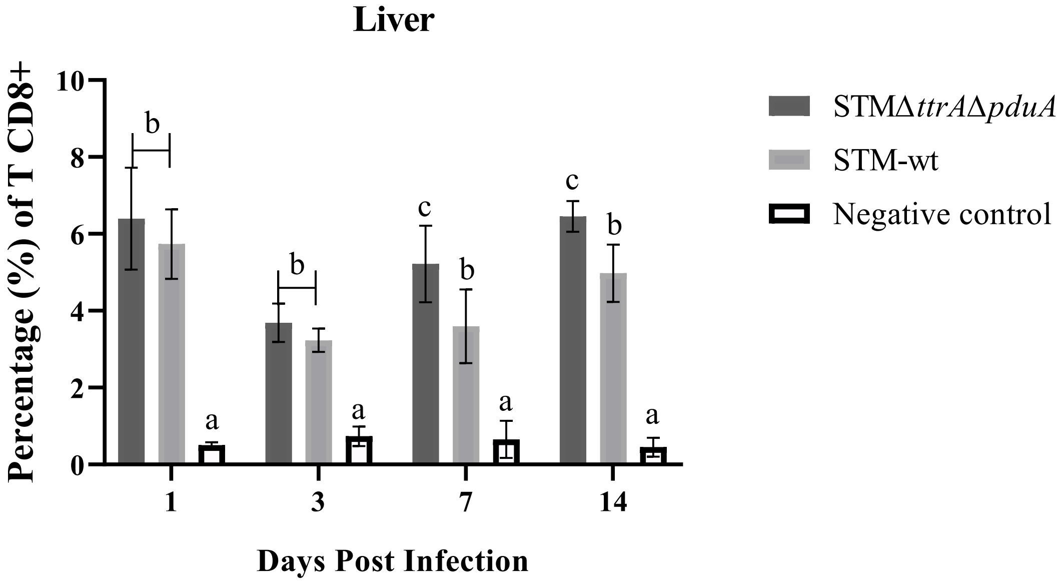

Liver
Percentage (%) of T CD8+
10
8
6
4
2
0
b
b
c
b
c
b
a
a
a
a
1
3
7
14
Days Post Infection
STMΔttrAΔpduA
STM-wt
Negative control

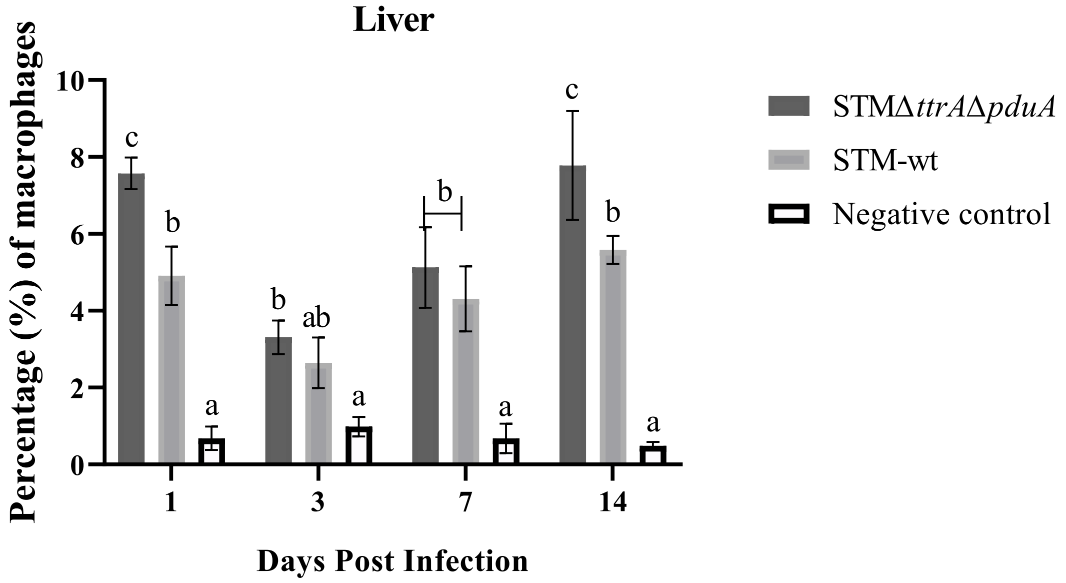
Liver
Percentage (%) of macrophages
10
8
6
4
2
0
c
b
a
b
ab
a
b
a
c
b
a
1
3
7
14
Days Post Infection
STMΔttrAΔpduA
STM-wt
Negative control

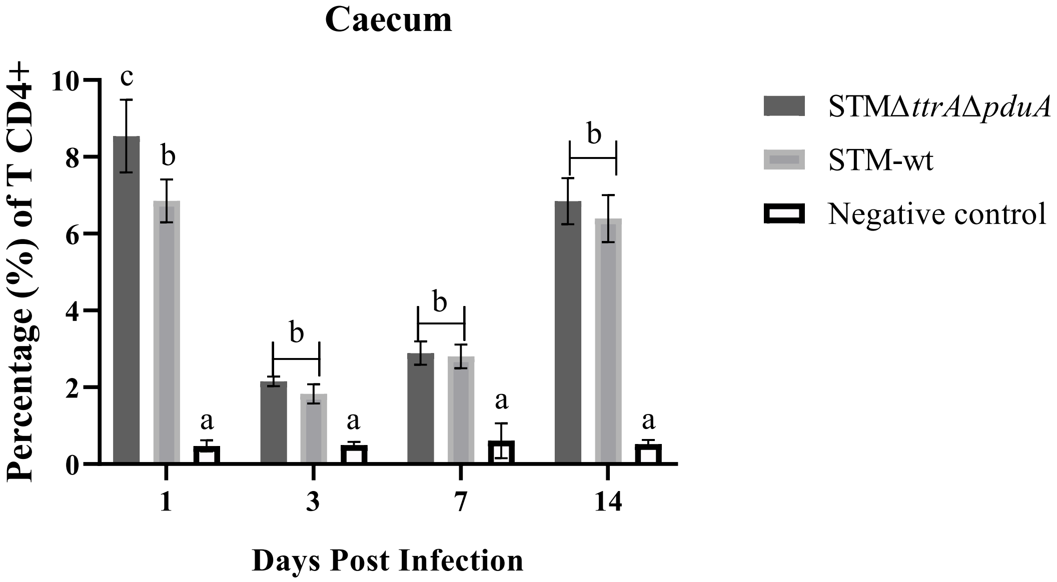
Caecum
Percentage (%) of T CD4+
10
8
6
4
2
0
c
b
a
b
a
b
a
b
a
1
3
7
14
Days Post Infection
STMΔ*ttrA*Δ*pduA*
STM-wt
Negative control

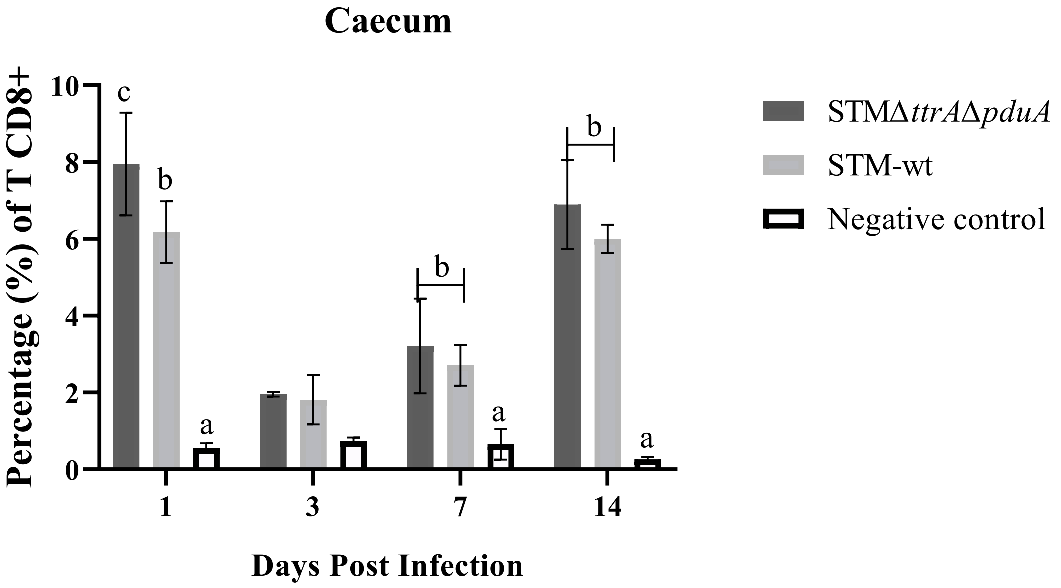

Caecum
Percentage (%) of T CD8+
10
8
6
4
2
0
c
b
a
b
a
b
a
1
3
7
14
Days Post Infection
STMΔ*ttrA*Δ*pduA*
STM-wt
Negative control

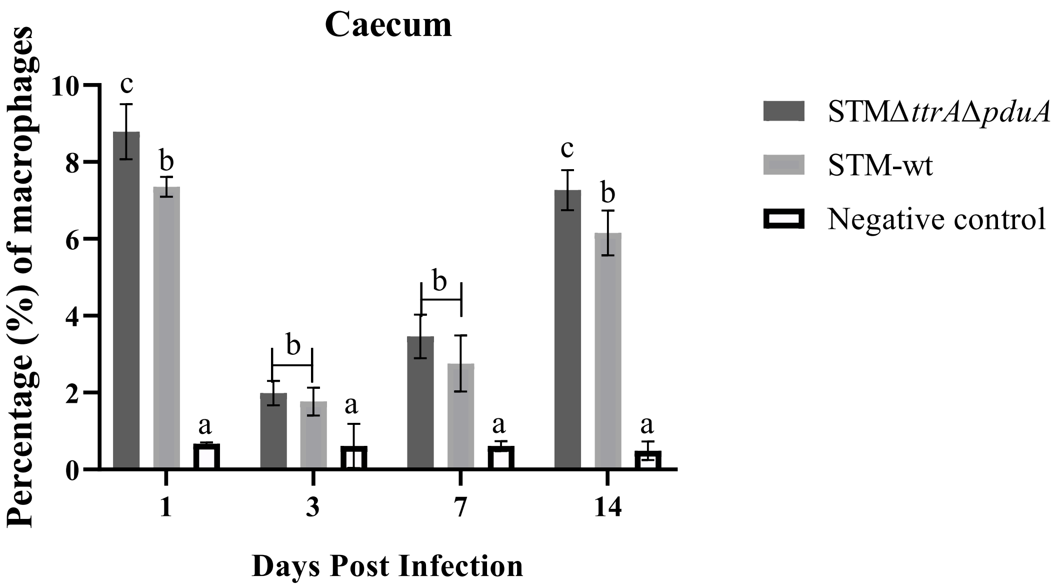

Caecum
Percentage (%) of macrophages
10
8
6
4
2
0
c
b
a
b
a
b
a
c
b
a
1
3
7
14
Days Post Infection
STMΔttrAΔpduA
STM-wt
Negative control

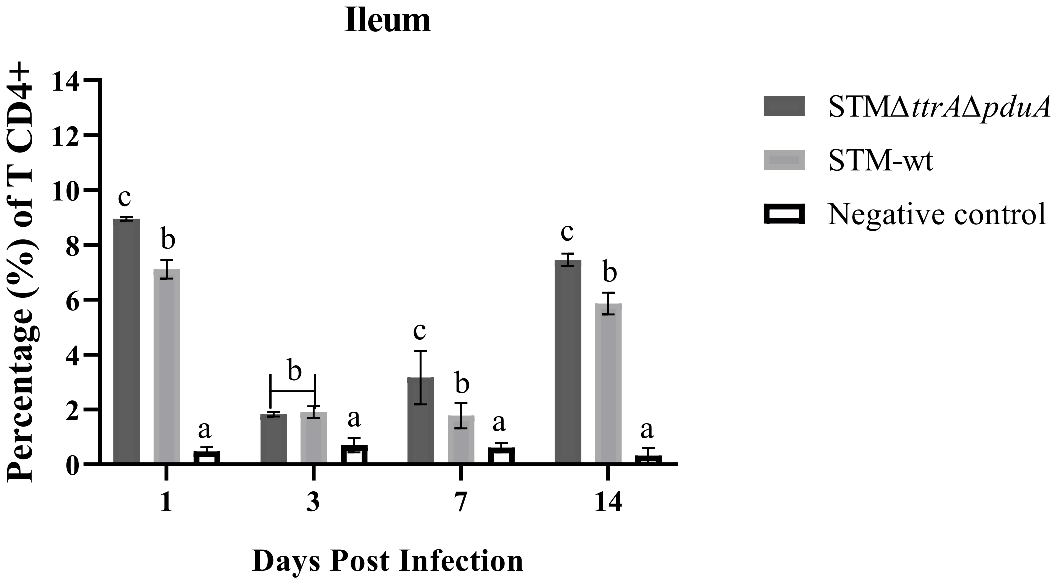

Ileum
Percentage (%) of T CD4+
14
12
10
8
6
4
2
0
c
b
a
b
a
c
b
a
c
b
a
1
3
7
14
Days Post Infection
STMΔ*ttrA*Δ*pduA*
STM-wt
Negative control

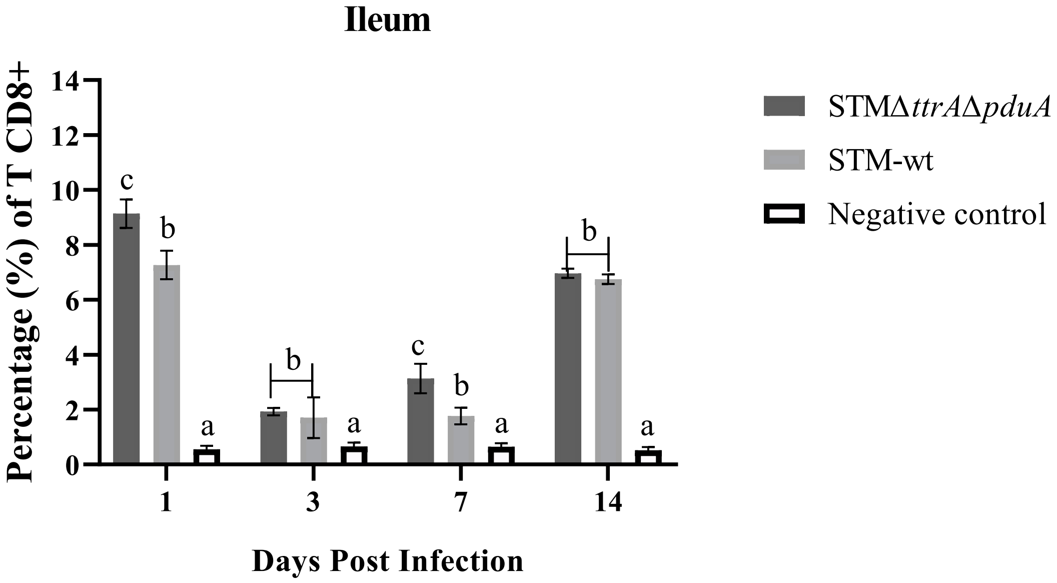
Ileum
Percentage (%) of T CD8+
14
12
10
8
6
4
2
0
c
b
a
b
a
c
b
a
b
a
1
3
7
14
Days Post Infection
STMΔttrAΔpduA
STM-wt
Negative control

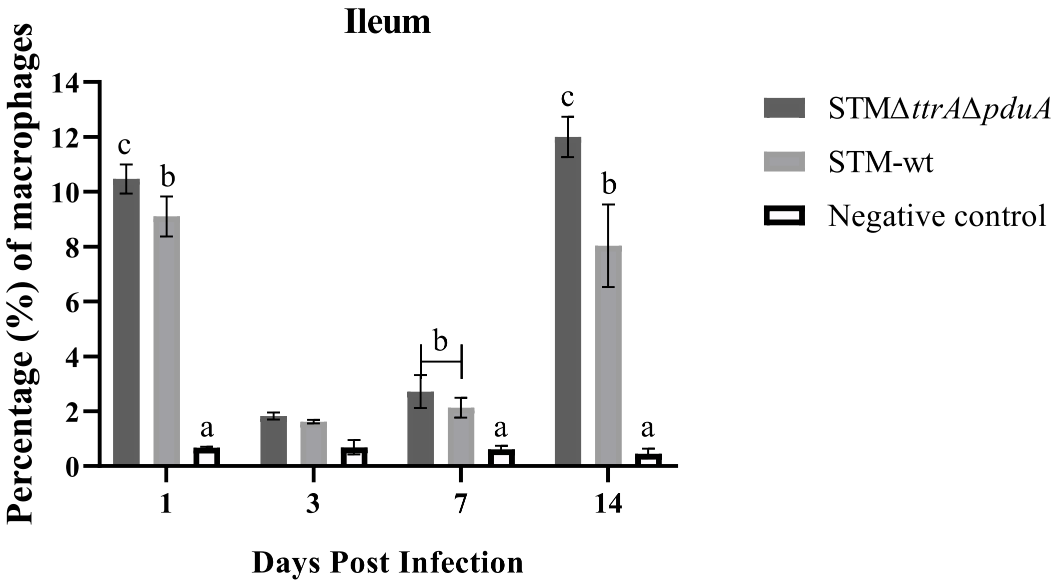
Ileum
Percentage (%) of macrophages
14
12
10
8
6
4
2
0
c
b
a
b
a
c
b
a
1
3
7
14
Days Post Infection
STMΔ*ttrA*Δ*pduA*
STM-wt
Negative control

Supplementary Figure S5. Percentage of the stained area by populations of lymphocytes T $CD4^+$ and $CD8^+$, and macrophages in the caecal tonsils, liver, caecum, and ileum of semi-heavy laying hens infected with *Salmonella* Typhimurium or *Salmonella* Typhimurium Δ*ttr*AΔ*pdu*A strains at different days post-infection. Different letters mean a significant statistical difference between challenged (mutant- and wild-type) and no challenged birds, in each of the days post-inoculation (dpi), by two-way ANOVA followed by Bonferroni multiple comparison test at 5% probability.

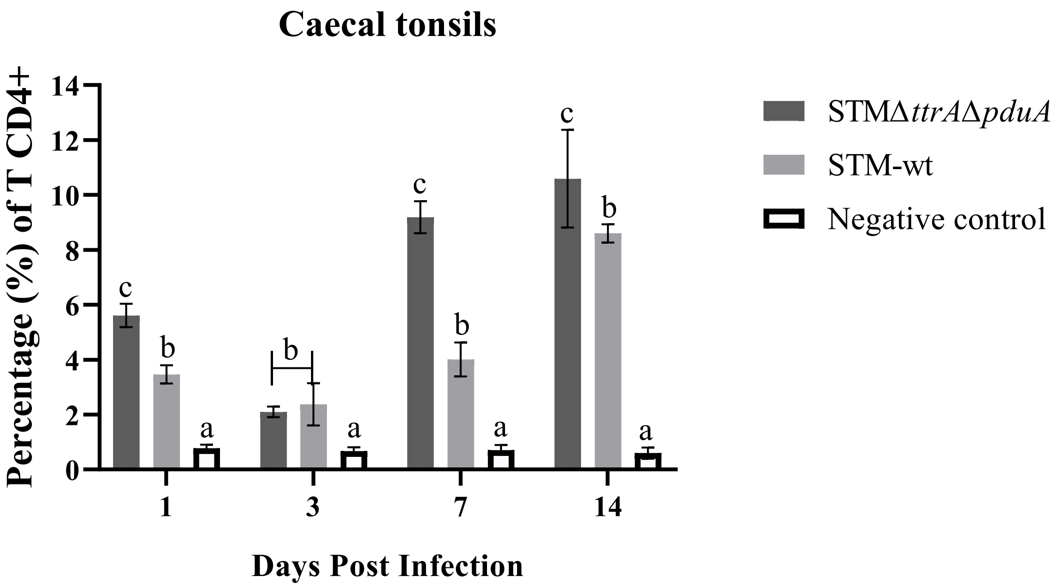

Caecal tonsils
Percentage (%) of T CD4+
14
12
10
8
6
4
2
0
c
b
a
b
a
c
b
a
c
b
a
1
3
7
14
Days Post Infection
STMΔttrAΔpduA
STM-wt
Negative control

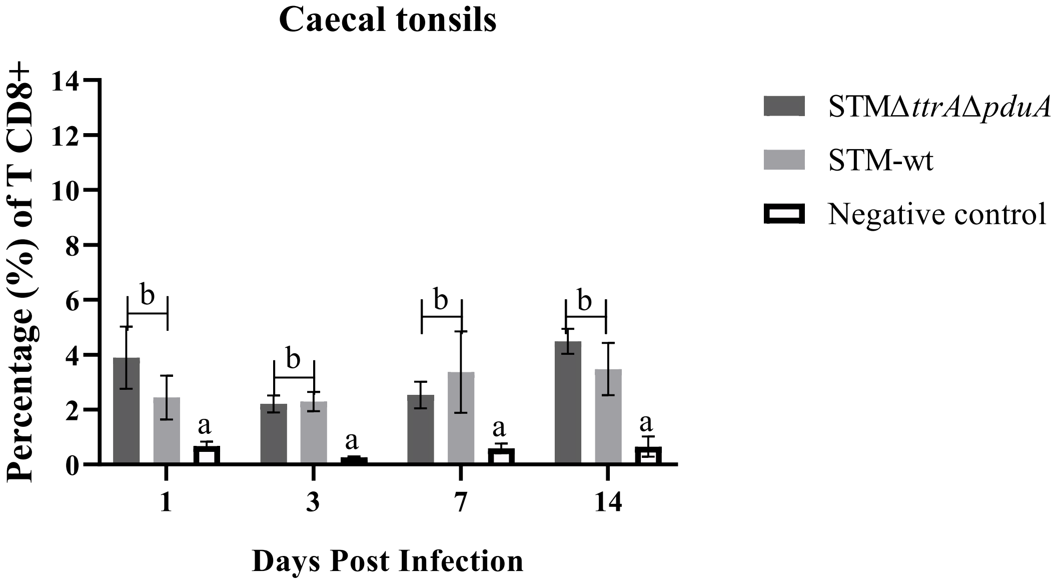
Caecal tonsils
Percentage (%) of T CD8+
14
12
10
8
6
4
2
0
b
a
1
3
7
14
Days Post Infection
STMΔttrAΔpduA
STM-wt
Negative control

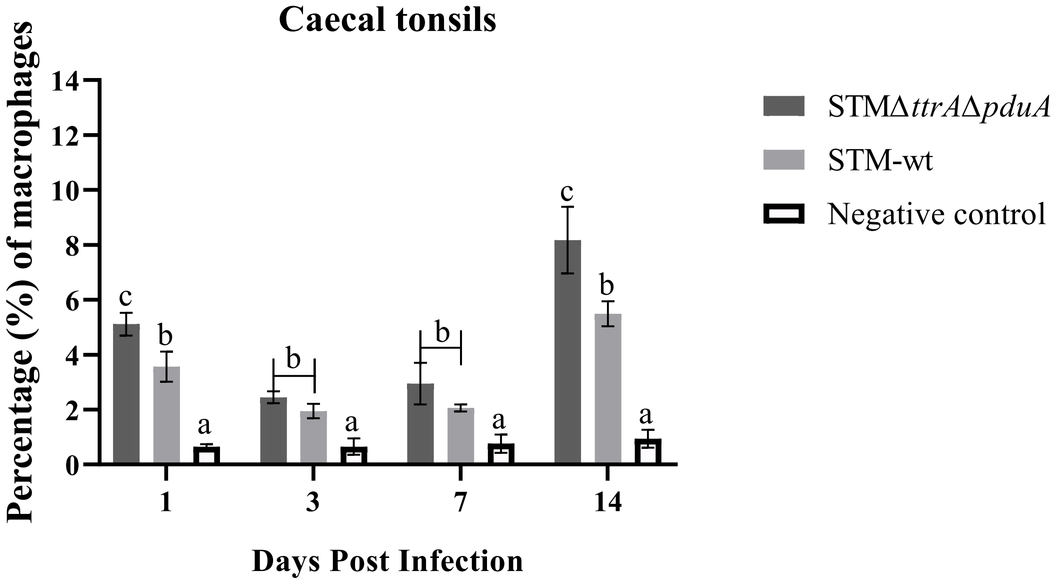
Caecal tonsils
Percentage (%) of macrophages
14
12
10
8
6
4
2
0
c
b
a
b
a
b
a
c
b
a
1
3
7
14
Days Post Infection
STMΔttrAΔpduA
STM-wt
Negative control

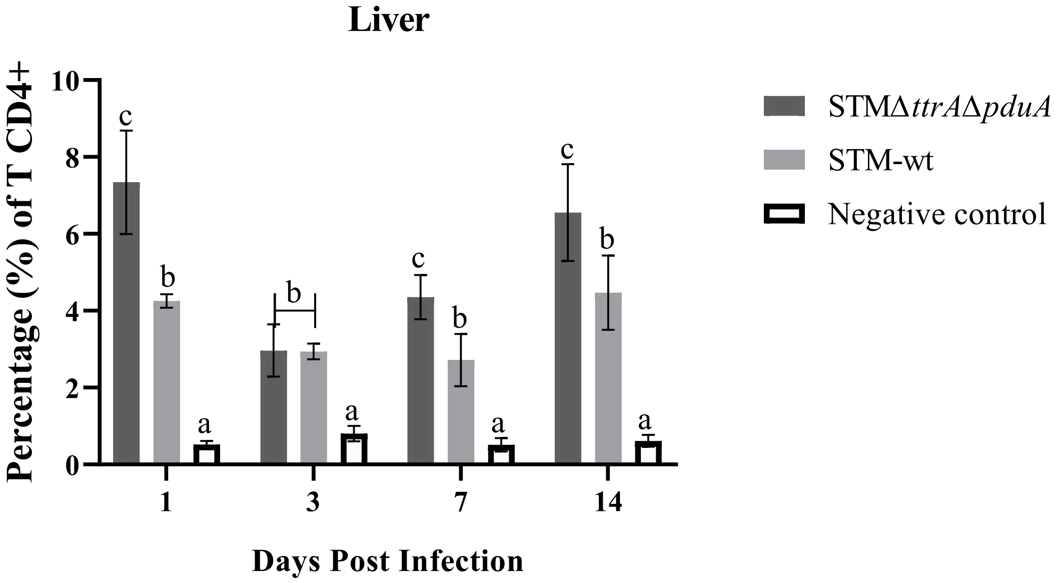
Liver
Percentage (%) of T CD4+
10
8
6
4
2
0
c
b
a
b
a
c
b
a
c
b
a
1
3
7
14
Days Post Infection
STMΔttrAΔpduA
STM-wt
Negative control

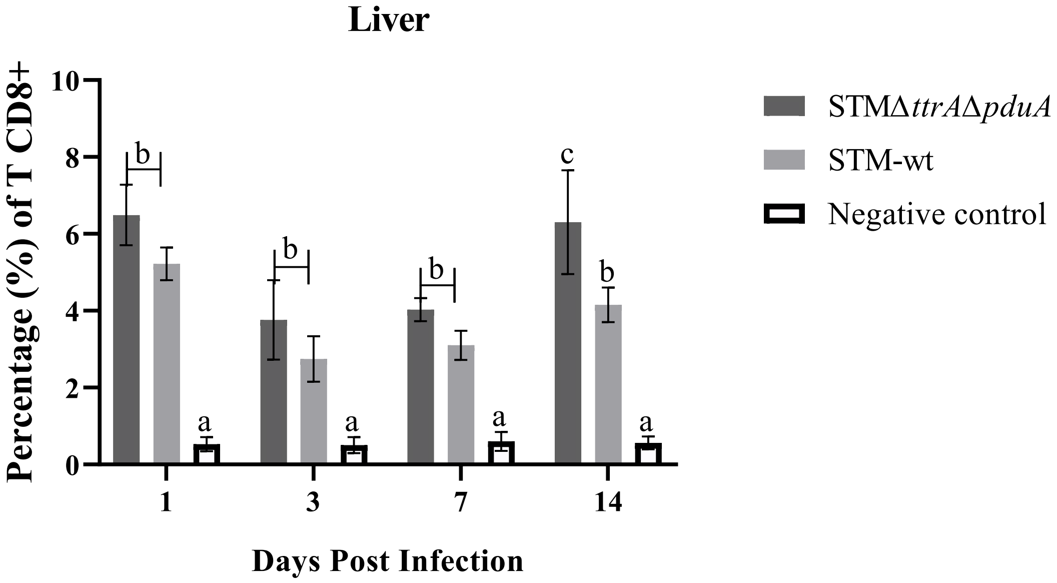
Liver
Percentage (%) of T CD8+
Days Post Infection
STMΔttrAΔpduA
STM-wt
Negative control
0
2
4
6
8
10
1
3
7
14
a
b
c

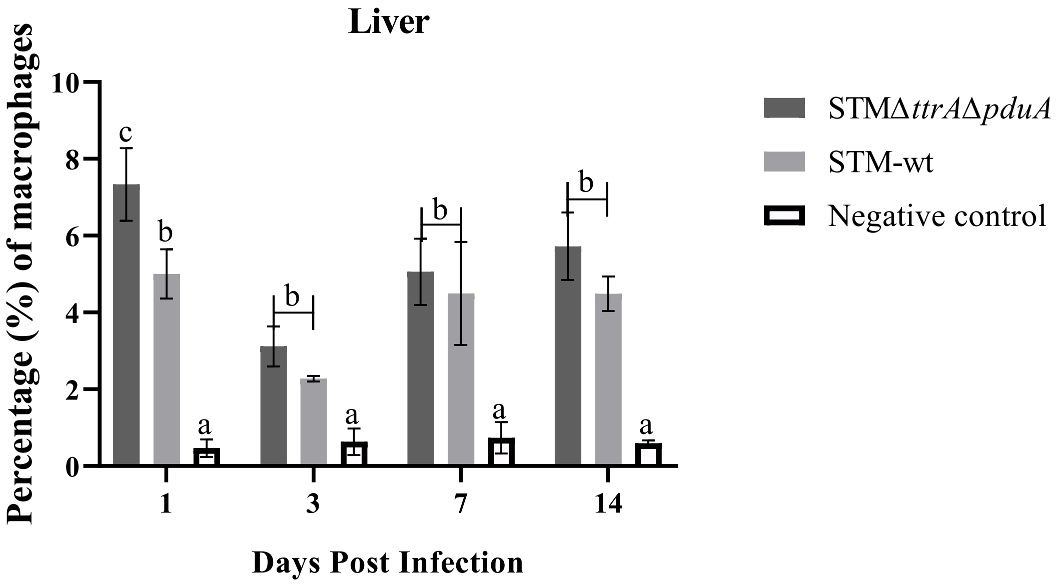

Liver
Percentage (%) of macrophages
10
8
6
4
2
0
c
b
a
b
a
b
a
b
a
1
3
7
14
Days Post Infection
STMΔttrAΔpduA
STM-wt
Negative control

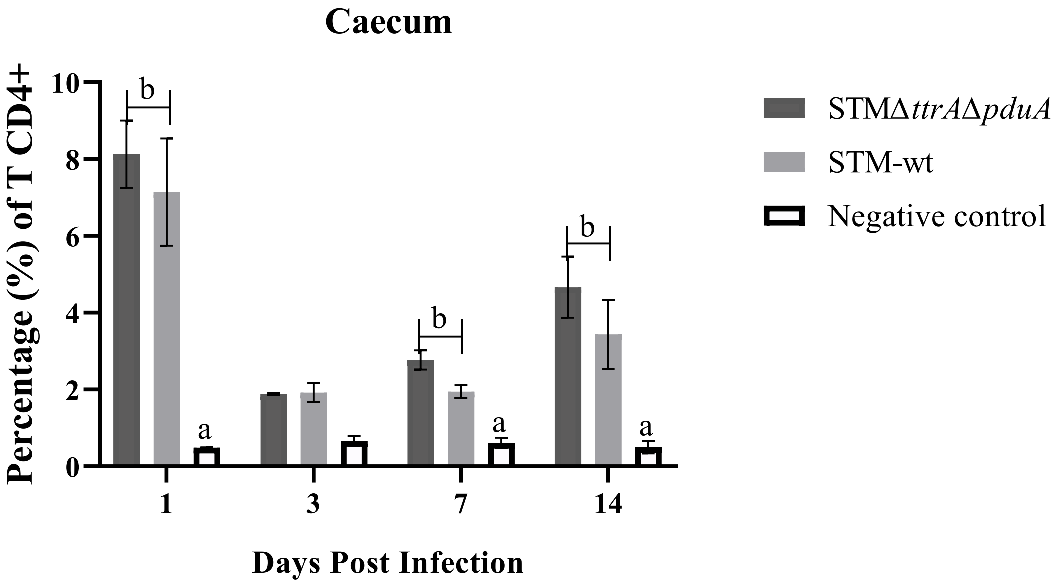

Caecum
Percentage (%) of T CD4+
10
8
6
4
2
0
b
b
b
a
a
a
1
3
7
14
Days Post Infection
STMΔttrAΔpduA
STM-wt
Negative control

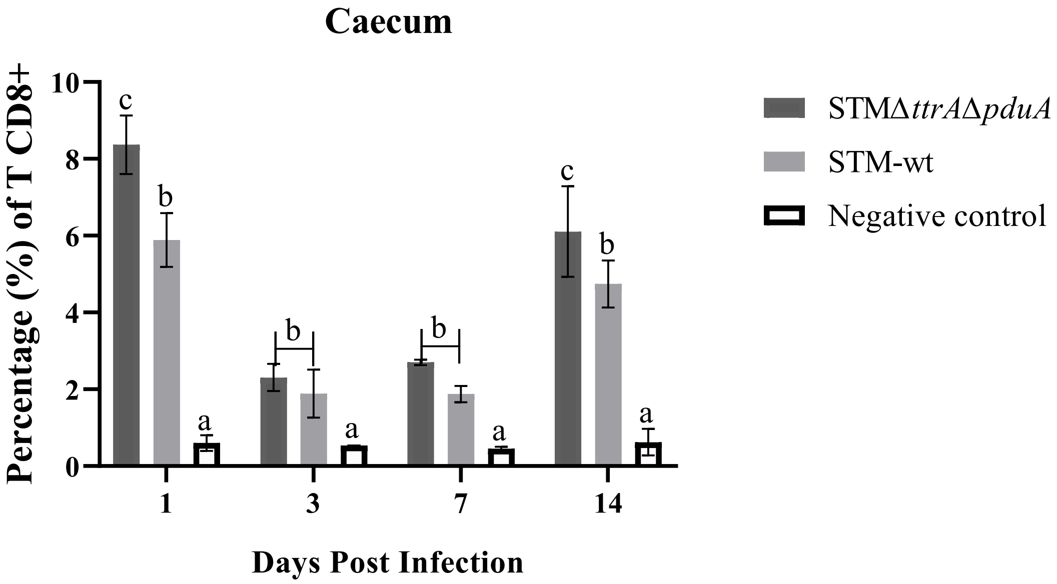
Caecum
Percentage (%) of T CD8+
10
8
6
4
2
0
c
b
a
b
a
b
a
c
b
a
1
3
7
14
Days Post Infection
STMΔttrAΔpduA
STM-wt
Negative control

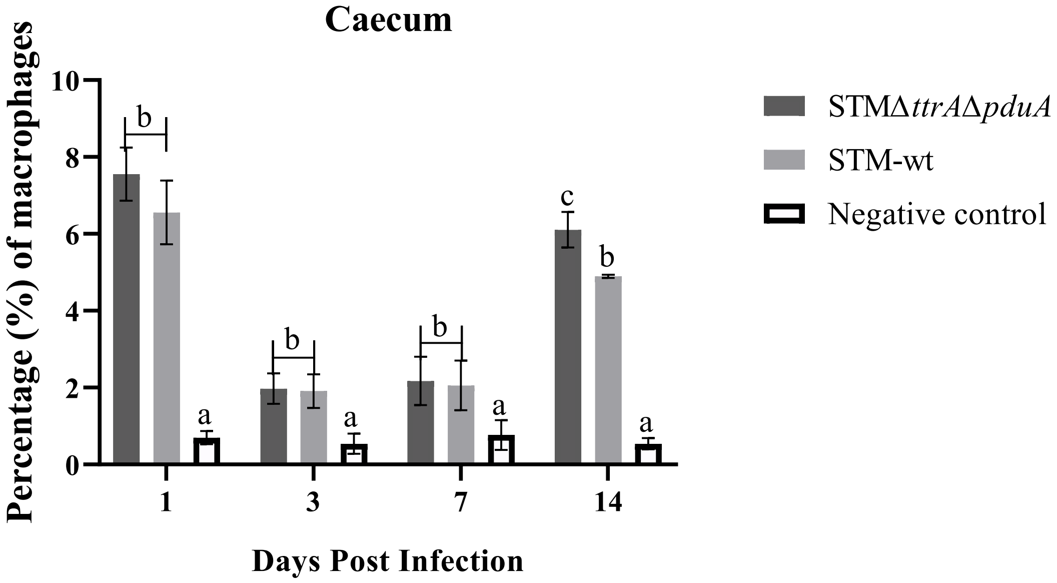

Caecum
Percentage (%) of macrophages
10
8
6
4
2
0
b
b
b
a
a
a
a
c
b
1
3
7
14
Days Post Infection
STMΔ*ttrA*Δ*pduA*
STM-wt
Negative control

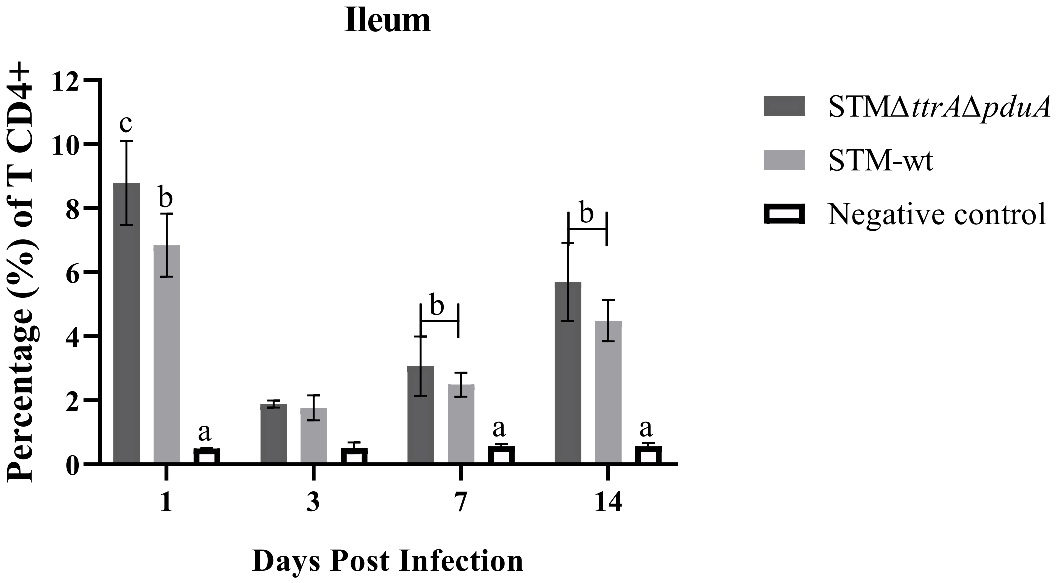
Ileum
Percentage (%) of T CD4+
12
10
8
6
4
2
0
c
b
a
b
a
b
a
1
3
7
14
Days Post Infection
STMΔttrAΔpduA
STM-wt
Negative control

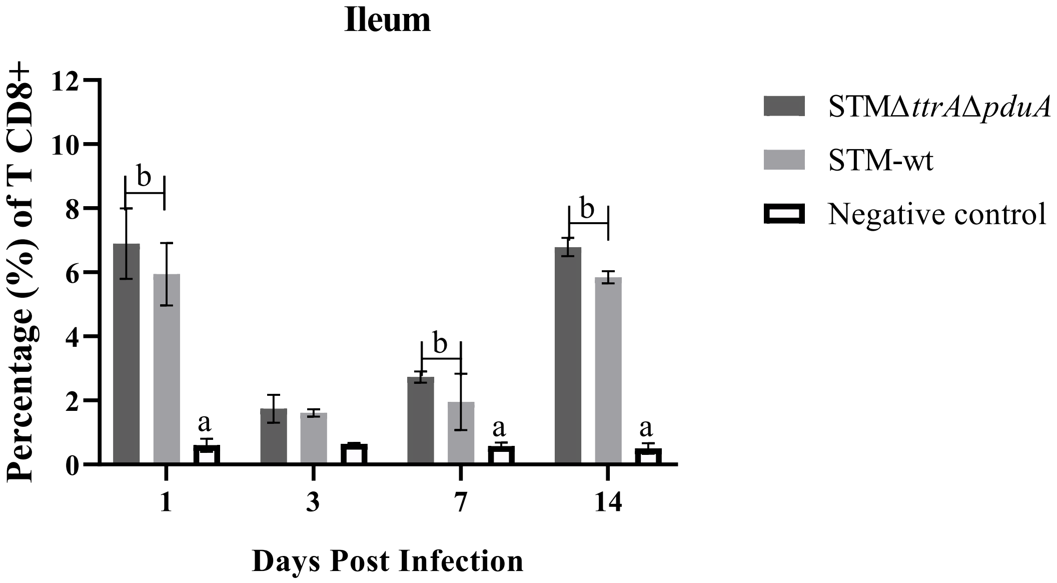
Ileum
Percentage (%) of T CD8+
12
10
8
6
4
2
0
b
b
b
a
a
a
1
3
7
14
Days Post Infection
STMΔ*ttrA*Δ*pduA*
STM-wt
Negative control

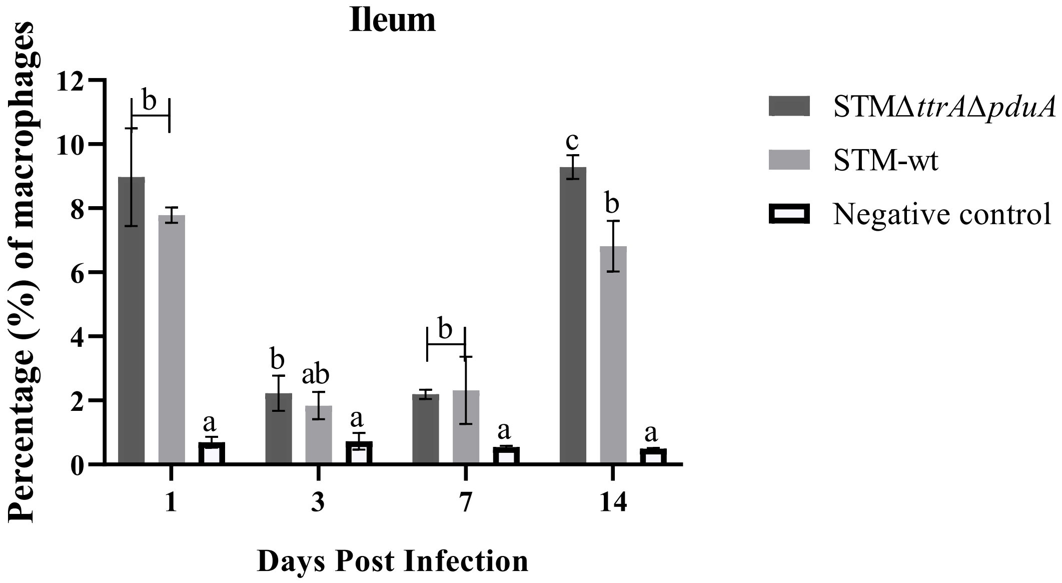

Ileum
Percentage (%) of macrophages
12
10
8
6
4
2
0
b
b
ab
a
a
b
a
c
b
a
1
3
7
14
Days Post Infection
STMΔttrAΔpduA
STM-wt
Negative control

Supplementary Figure S6. Percentage of the stained area by populations of lymphocytes T $CD4^+$ and $CD8^+$, and macrophages in the caecal tonsils, liver, caecum, and ileum of light laying hens infected with *Salmonella* Typhimurium or *Salmonella* Typhimurium Δ*ttr*AΔ*pdu*A strains at different days post-infection. Different letters mean a significant statistical difference between challenged (mutant- and wild-type) and no challenged birds, in each of the days post-inoculation (dpi), by two-way ANOVA followed by Bonferroni multiple comparison test at 5% probability.

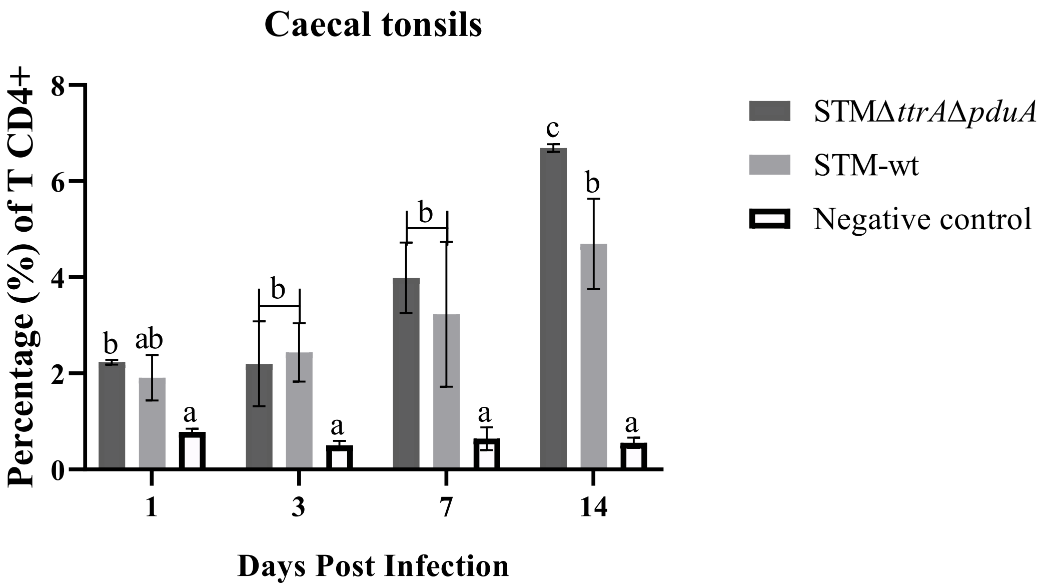

Caecal tonsils
Percentage (%) of T CD4+
8
6
4
2
0
b
ab
a
b
a
b
a
c
b
a
1
3
7
14
Days Post Infection
STMΔttrAΔpduA
STM-wt
Negative control

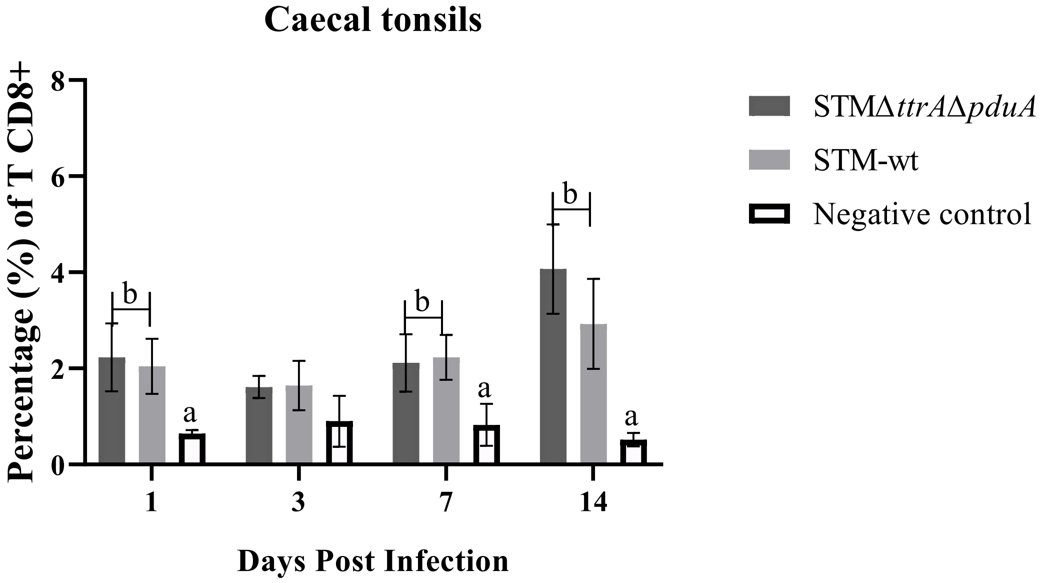
Caecal tonsils
Percentage (%) of T CD8+
8
6
4
2
0
b
b
b
a
a
a
1
3
7
14
Days Post Infection
STMΔ*ttrA*Δ*pduA*
STM-wt
Negative control

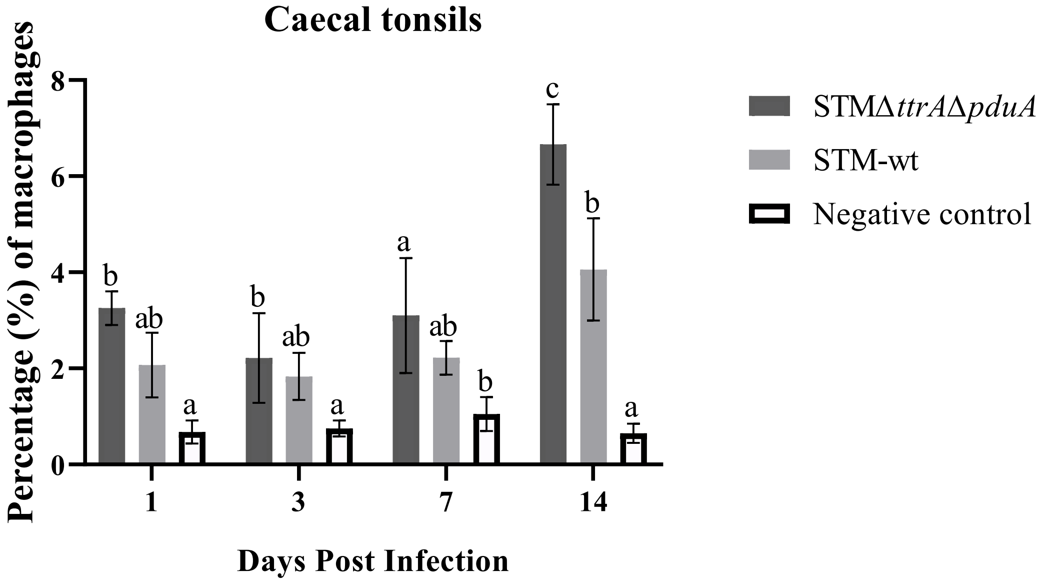

Caecal tonsils
Percentage (%) of macrophages
8
6
4
2
0
b
ab
a
b
ab
a
a
ab
b
c
b
a
1
3
7
14
Days Post Infection
STMΔttrAΔpduA
STM-wt
Negative control

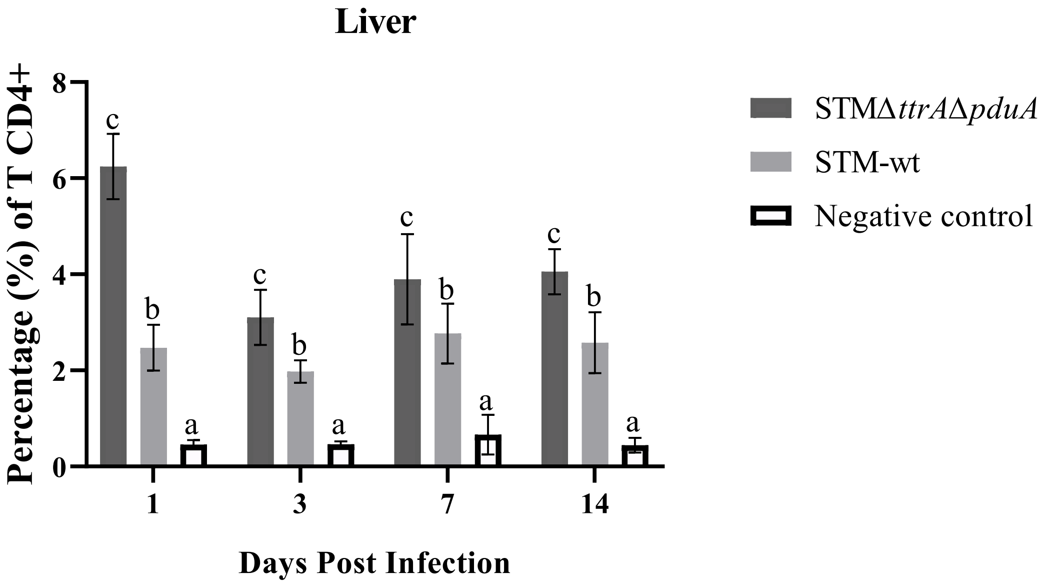

Liver
Percentage (%) of T CD4+
8
6
4
2
0
c
b
a
1
3
7
14
Days Post Infection
STMΔttrAΔpduA
STM-wt
Negative control

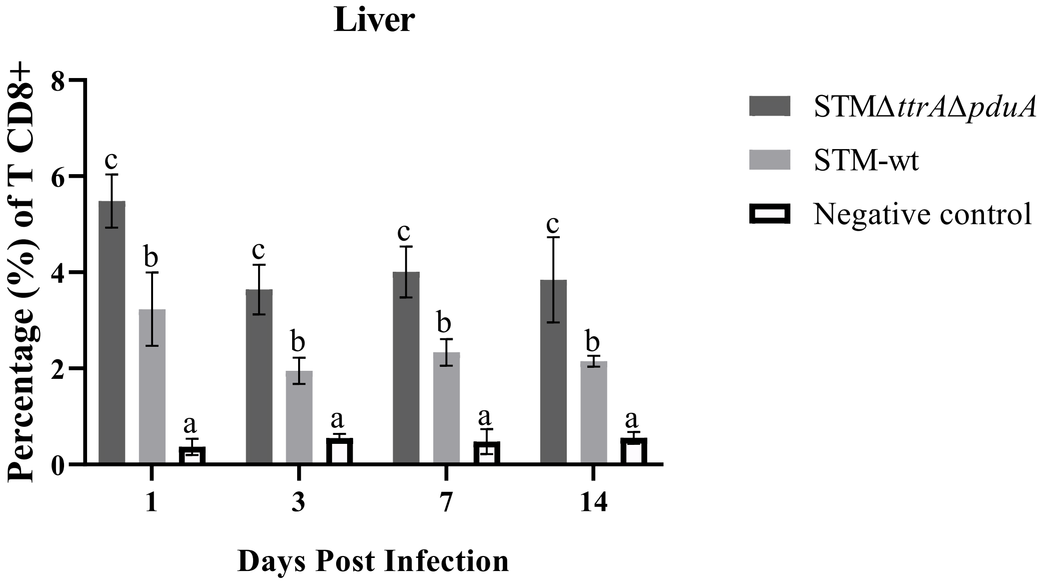
Liver
Percentage (%) of T CD8+
8
6
4
2
0
c
b
a
1
3
7
14
Days Post Infection
STMΔ*ttrA*Δ*pduA*
STM-wt
Negative control

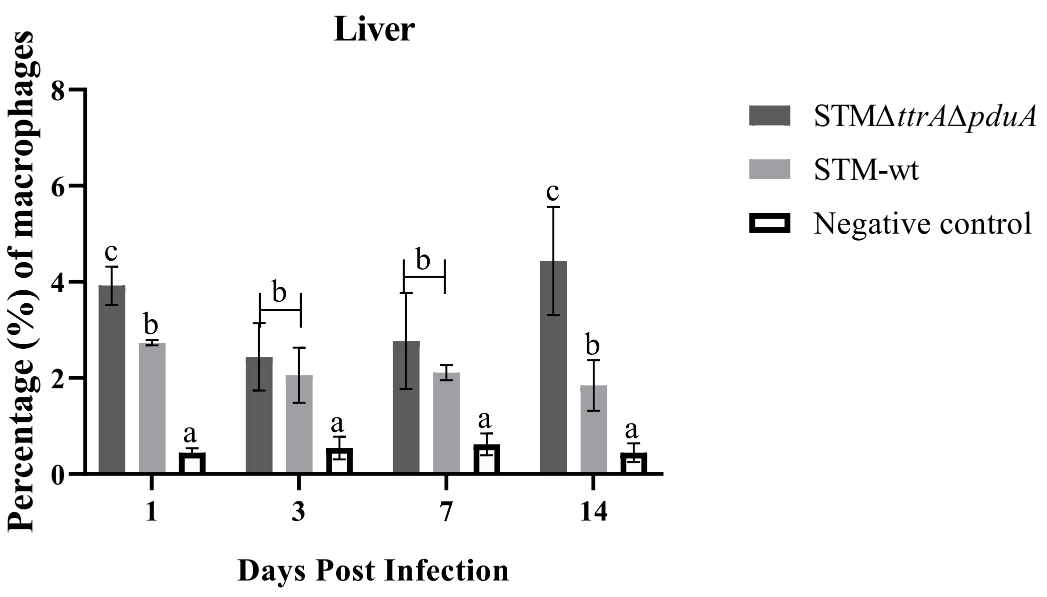

Liver
Percentage (%) of macrophages
0
2
4
6
8
c
b
a
b
a
b
a
c
b
a
1
3
7
14
Days Post Infection
STMΔ*ttrA*Δ*pduA*
STM-wt
Negative control

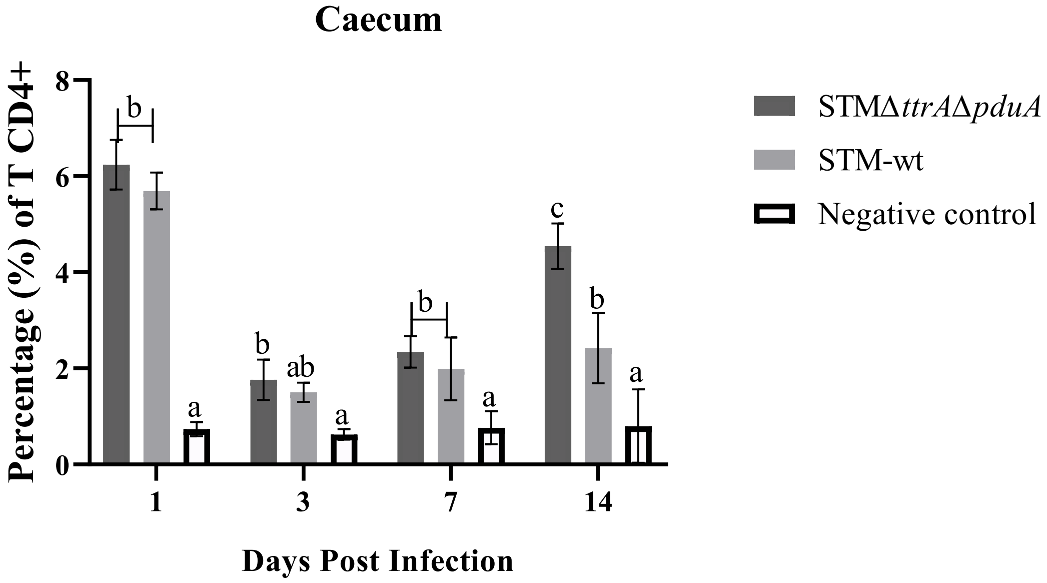

Caecum
Percentage (%) of T CD4+
8
6
4
2
0
b
b
ab
a
a
a
b
a
c
b
a
1
3
7
14
Days Post Infection
STMΔ*ttrA*Δ*pduA*
STM-wt
Negative control

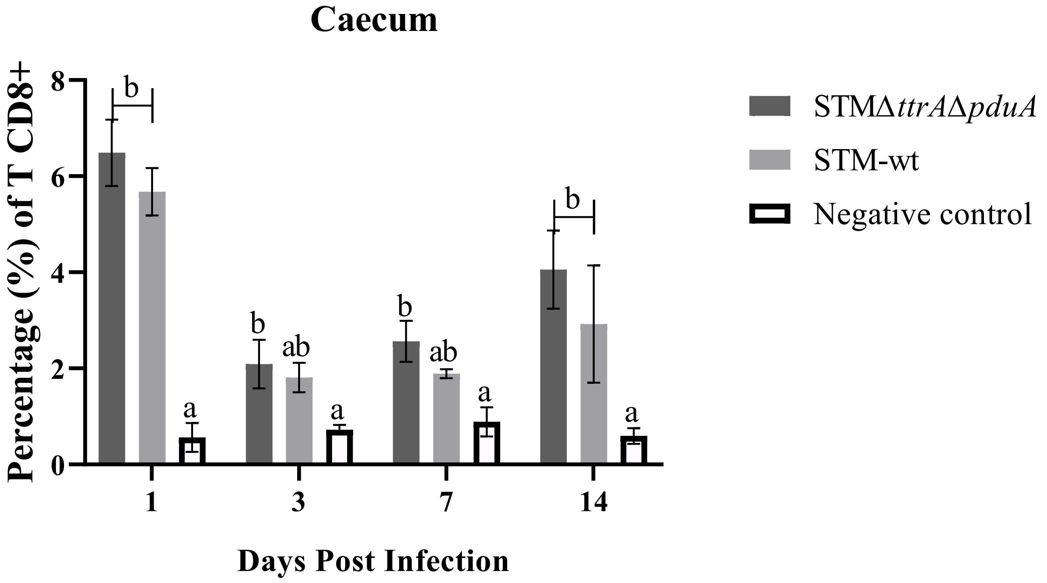
Caecum
Percentage (%) of T CD8+
Days Post Infection
STMΔ*ttrA*Δ*pduA*
STM-wt
Negative control
0
2
4
6
8
1
3
7
14
b
a
ab
b
a
b
ab
a
b
a

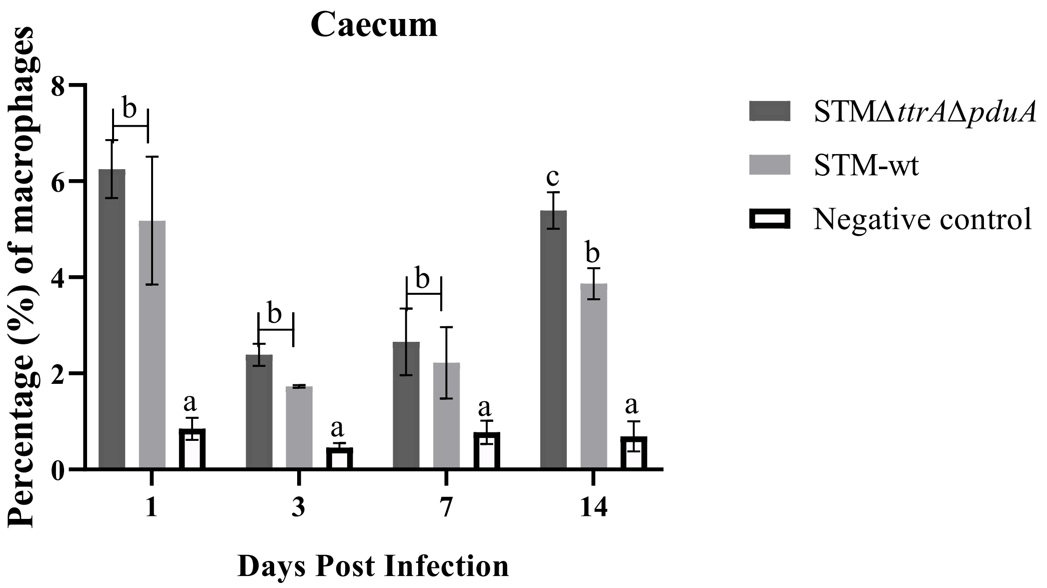

Caecum
Percentage (%) of macrophages
8
6
4
2
0
b
b
b
c
b
a
a
a
a
1
3
7
14
Days Post Infection
STMΔttrAΔpduA
STM-wt
Negative control

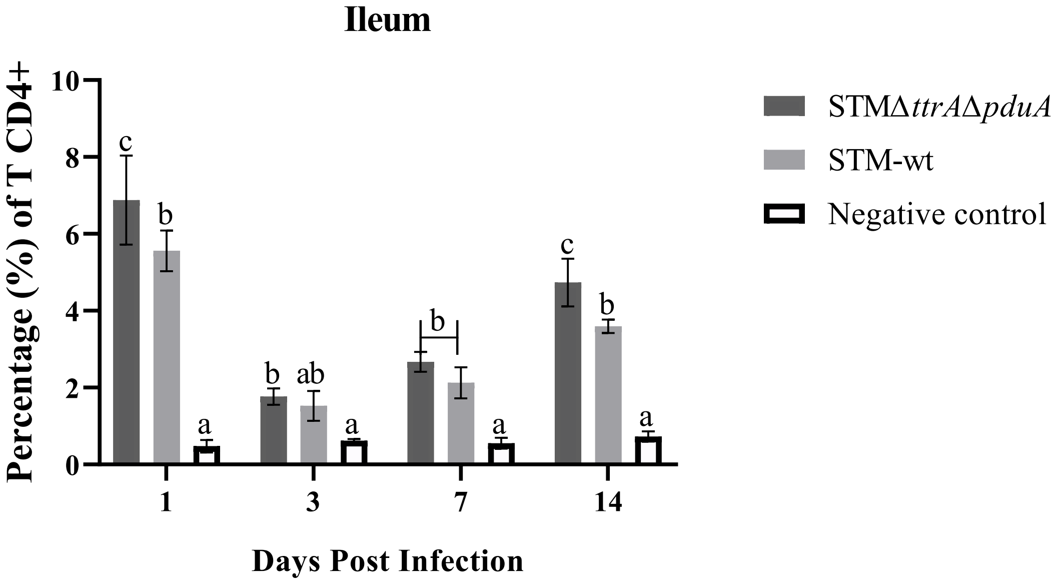

Ileum
Percentage (%) of T CD4+
0
2
4
6
8
10
c
b
a
b
ab
a
b
a
c
b
a
1
3
7
14
Days Post Infection
STMΔ*ttrA*Δ*pduA*
STM-wt
Negative control

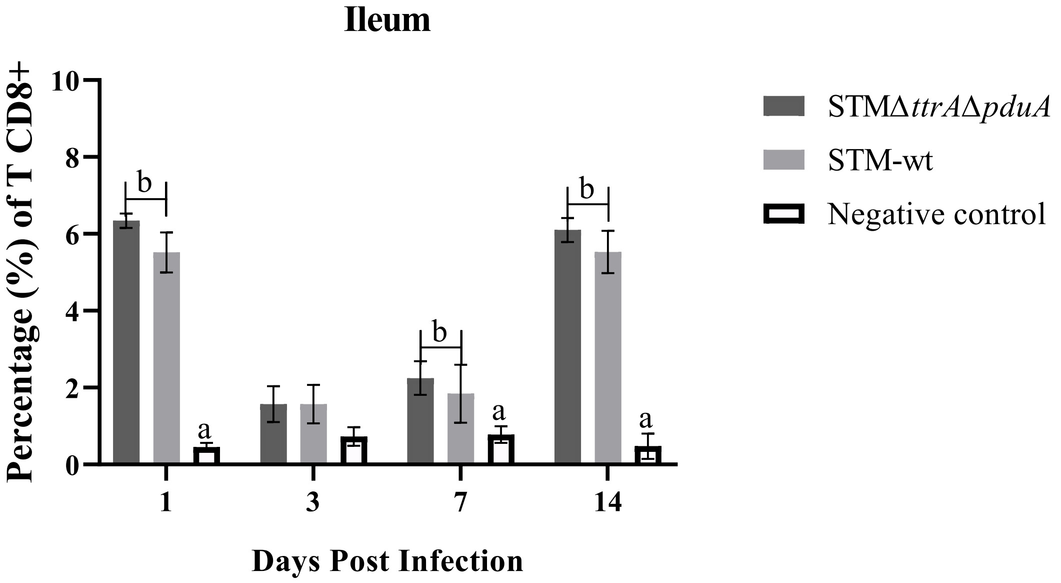
Ileum
Percentage (%) of T CD8+
10
8
6
4
2
0
b
a
b
a
b
a
1
3
7
14
Days Post Infection
STMΔ*ttrA*Δ*pduA*
STM-wt
Negative control

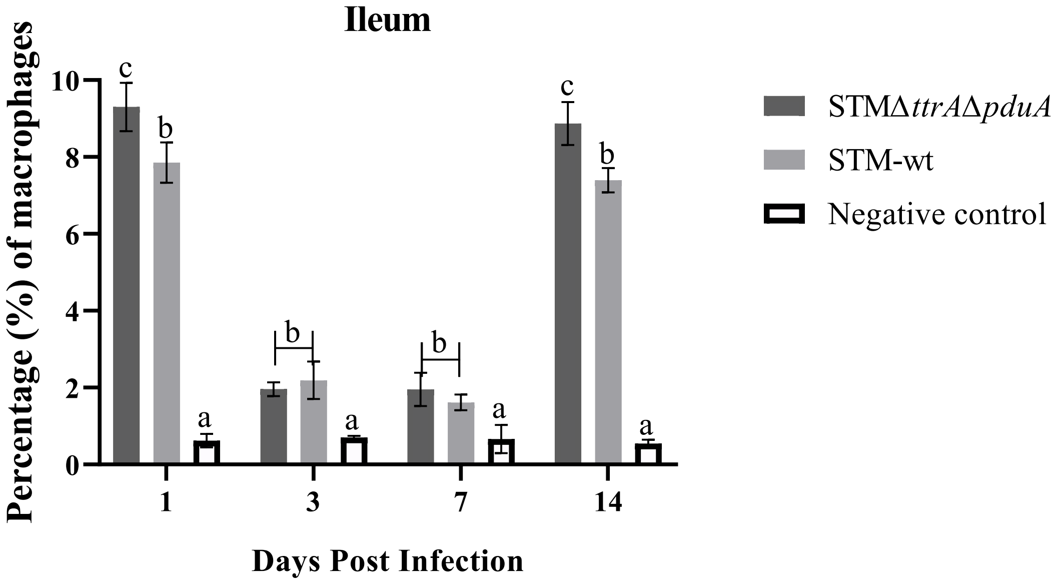

Ileum
Percentage (%) of macrophages
10
8
6
4
2
0
c
b
a
b
a
b
a
c
b
a
1
3
7
14
Days Post Infection
STMΔ*ttrA*Δ*pduA*
STM-wt
Negative control
